# A Critical Review on Classified Excipient Sodium-Alginate-Based Hydrogels: Modification, Characterization, and Application in Soft Tissue Engineering

**DOI:** 10.3390/gels9050430

**Published:** 2023-05-22

**Authors:** Rishav Sharma, Rishabha Malviya, Sudarshan Singh, Bhupendra Prajapati

**Affiliations:** 1 Department of Pharmacy, School of Medical and Allied Sciences, Galgotias University, Greater Noida 203201, India; srishav45@gmail.com; 2 Department of Pharmaceutical Sciences, Faculty of Pharmacy, Chiang Mai University, Chiang Mai 50200, Thailand; sudarshansingh83@hotmail.com; 3 Shree S. K. Patel College of Pharmaceutical Education and Research, Ganpat University, Kherva 384012, India

**Keywords:** sodium alginate, hydrogels, tissue engineering, characterization

## Abstract

Alginates are polysaccharides that are produced naturally and can be isolated from brown sea algae and bacteria. Sodium alginate (SA) is utilized extensively in the field of biological soft tissue repair and regeneration owing to its low cost, high biological compatibility, and quick and moderate crosslinking. In addition to their high printability, SA hydrogels have found growing popularity in tissue engineering, particularly due to the advent of 3D bioprinting. There is a developing curiosity in tissue engineering with SA-based composite hydrogels and their potential for further improvement in terms of material modification, the molding process, and their application. This has resulted in numerous productive outcomes. The use of 3D scaffolds for growing cells and tissues in tissue engineering and 3D cell culture is an innovative technique for developing in vitro culture models that mimic the in vivo environment. Especially compared to in vivo models, in vitro models were more ethical and cost-effective, and they stimulate tissue growth. This article discusses the use of sodium alginate (SA) in tissue engineering, focusing on SA modification techniques and providing a comparative examination of the properties of several SA-based hydrogels. This review also covers hydrogel preparation techniques, and a catalogue of patents covering different hydrogel formulations is also discussed. Finally, SA-based hydrogel applications and future research areas concerning SA-based hydrogels in tissue engineering were examined.

## 1. Introduction

**Figure 1:**
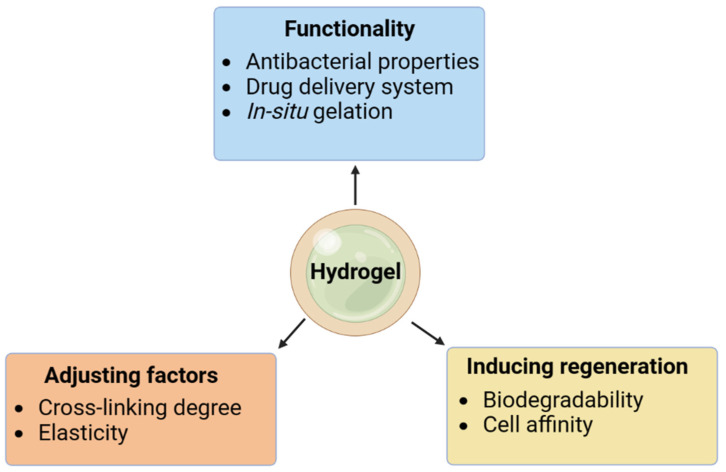
Parameters for optimal hydrogels.

The majority of human organs are made up of soft tissues, and since these tissues can regenerate, patients will have a far lower chance of needing an organ transplant [1]. However, regenerating soft tissues is challenging because of their multicellular population and complicated stratified structural properties [2,3]. Hydrogels possess significant potential in the realm of soft tissue engineering despite sharing structural and physicochemical features with the natural extracellular matrix (ECM) [4]. This involves the building of blood arteries, the heart, skin, nerves, muscles, and the liver. Hydrogels’ use in tissue engineering has grown exponentially since the development of bioprinting technology. Scaffolds of systematically complex structures can now be manufactured, and critical dimensional parameters, such as scaffold porosity and pore size, can be effectively controlled due to bioprinting’s ability to deposit different biomaterials (cells, growth factors, hydrogels, etc.) precisely and on demand in time and space. Consequently, the biomechanical development of tissue-engineered functional organs receives new hope with the combination of hydrogel-based biomaterials with 3D bioprinting technology. Many impressive advances have been made in the regeneration and restoration of soft tissues using 3D bioprinting hydrogel technology, which is now being used by an increasing number of academic researchers. Regenerative medicine is an emerging field; however, it has made remarkable progress in recent years. Its practices increase cell proliferation and differentiation and work to stimulate the body’s innate healing mechanisms. Multiple types of cells linked to tissue regeneration are delivered to the damaged location, which aids in tissue repair that goes beyond the self-healing potential. To thrive and perform their functions, cells need to communicate with one another and with the ECM (the extracellular matrix). The cells successfully express their biological roles in the body; however, the body requires tissue engineering materials that mimic the extracellular matrix (ECM) during tissue regeneration. In the field of tissue engineering, among the most significant difficulties is the development of solutions that can restore native tissue-like forms and functions. To optimize the conditions for tissue repair, researchers have undertaken several efforts to learn more about the relationships between cells, scaffolds, and bioactive chemicals [5]. Hydrogels have received a lot of attention for their use in a wide variety of applications, especially in soft tissues, because of their adaptability in terms of synthesis and functionalization for regulated biodegradation and mechanical characteristics. Hydrogels’ three-dimensional network design may be easily adjusted to achieve the required physical properties by measuring and controlling such factors as crosslinking density, elastic modulus, and degradation rate [6,7]. Thus, the degradation time can be set to correspond with the pace at which the target tissue is being regenerated (Figure 1), and the form and flexibility can be matched to the target tissue. The biocompatibility of the material allows for additional bioactivity to be introduced via the combination of functions and the conjugation of several bioactive compounds. These features are desirable because they make it possible to build materials in line with the stated regeneration strategy for each tissue. More and more hydrogels are being made from organically generated polymers because of their biocompatibility and valuable biofunctions [7]. Recent years have seen the increasing use of cell culture systems as scaffolds in which cells are added, in conjunction with 3D materials, as a method of acquiring even more complex regeneration tissues with the addition of growth factors, biological signal factors (i.e., peptides), and medicines. Sodium alginate (SA) has emerged as a popular hydrogel and is now considered a promising material for use in soft tissue scaffolds [8,9]. Sodium alginate is a natural polysaccharide that originates from brown algae kelp or Sargassum. The molecules that make up the compound comprise mannuronic acid and guluronic acid, which are bonded together by (1→4) bonds. Sodium alginate also has the benefits of being inexpensive, non-cytotoxic, simple to work with, and fast to gel [10,11]. The produced hydrogels share numerous structural and physicochemical characteristics with the natural extracellular matrix [12], which renders them promising for enclosing cells in the highly hydrated three-dimensional environment required for the formation and repair of soft tissue architecture. However, SA has some disadvantages in the field of soft tissue engineering: (i) due to its scaffold’s lackluster mechanical qualities, it cannot offer adequate mechanical support in a demanding setting. (ii) it suffers from a deterioration that occurs gradually and cannot be stopped; (iii) it is unable to engage with cells and provide adequate adhesion sites for cells [13,14]. Since sodium alginate hydrogels are used for tissue engineering, the absence of these flaws is crucial. Many unique physical, biological, and chemical characteristics of the substance (including its mechanical stiffness, swelling, disintegration, cell adherence, and its combination with bioactive compounds to provide a delayed release of growth factors) are the targets of physical and chemical modification techniques used by researchers [15,16]. This allows sodium alginate to better meet the diverse functional requirements of tissue engineering.

## 2. Natural Biological Substrates

Agarose, collagen, gelatin, chitosan, carrageenan, and fibrin are just a few of the natural biomaterials that have been successfully used in tissue engineering [17,18]. These biopolymers are advantageous because of their high compatibility, inherent non-toxicity, and strong cell adherence. By mixing sodium alginate with various other natural biomaterials that are highly biocompatible, cell migration and proliferation can be accelerated. Natural biomaterials are being incorporated into the sodium alginate hydrogel to enhance its mechanical properties. These natural materials work by developing hydrogen bonds between the hydroxyl and carboxyl groups of sodium alginate molecules, a process that in turn strengthens the hydrogel’s three-dimensional interconnected structure. Sodium alginate is frequently mixed with different biological materials with excellent biological characteristics in the field of soft tissue engineering to improve the physical and chemical properties of the materials and to address the requirements of a wide range of applications. These biomaterials include agarose [19], chitosan [20,21], hyaluronic acid [22,23], gelatin [24,25], collagen [26,27], fibrin [28], and others. Hydrogels are widely used in biomedical applications, with SA/gelatin composite hydrogels enjoying special popularity. The bioactive amino acid residues that increase cell adhesion sites and trigger cell adhesion are not contained in sodium alginate, but they are found in gelatin produced from collagen [29,30]. Gelatin, additionally, has reversible thermodynamic properties, and its modulus and viscosity are enhanced by the formation of a triple helix structure at low temperatures [31,32] and may significantly enhance the printing qualities of hybrid hydrogels. The gelatin in combination with sodium alginate prevents spontaneous thermal gelation [33], which enables a low viscosity to be maintained at room temperature. The printed material’s biomechanical and biological qualities can be tailored by altering the sodium alginate and gelatin levels in the composite hydrogel. When tested for printability, mechanical strength, and cellular activity, a mixture of 7% SA and 8% gelatin improved each component individually [27]. Capgel hydrogels are generated artificially from sodium alginate and gelatin, and they feature regular, cylinder-shaped microchannels. Microvascularization can be supported, and directional cell growth can be induced, using the scaffolds created when Capgel hydrogels are chopped into small pieces and printed using a syringe. Sodium alginate hydrogels’ mechanical characteristics can be enhanced by incorporating gelatin (which provides adjustable bio-functioning) [26], although this particular enhancement is insufficient to meet the criteria of a mechanical property [31]. In addition to these natural biopolymers, there is currently a considerable amount of curiosity in a recently created chemically processed biomaterial called a decellularized extracellular matrix. Sodium alginate differentiates itself from biomaterials because it improves with the addition of a decellularized extracellular matrix. Bioinks formulated with dECM have the potential to increase the yield strength and enhance cell protection during 3D printing [31,34]. This is due to the numerous components of dECM, such as collagen, fibronectin, and biopolysaccharides. In addition, while sodium alginate on its own does not stimulate blood vessel growth, dECM does. A sodium alginate/decellularized extracellular matrix has been shown to stimulate the growth and development of smooth muscle cells (SMC) and epithelial progenitor cells (EPC) in human bronchial tissue [32]. This sodium alginate/decellularized extracellular matrix bioink shows great promise as a future bioink for human transplantation [35,36].

## 3. Hydrogel Preparation

**Figure 2:**
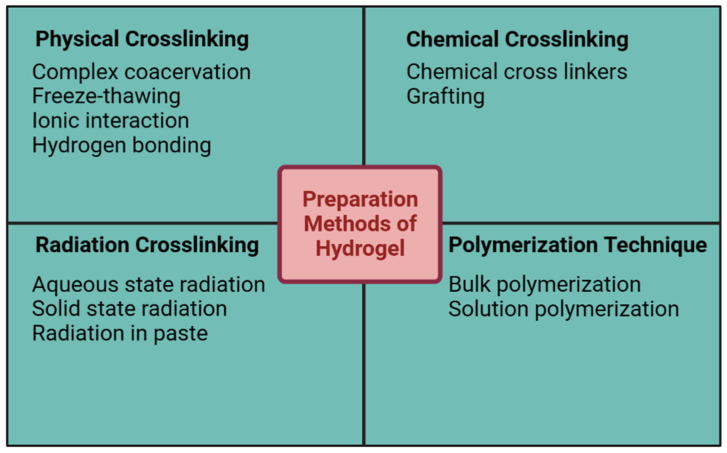
Different methods for the preparation of hydrogel.

Hydrogels are often made with hydrophilic monomers, which are used to create a crosslinked network capable of absorbing water. The gel point, or the moment of transition of the polymer mixture from a sol state to a gel state (the gelation manifestation), can be identified for each hydrogel using rheological studies [37]. There are numerous approaches to hydrogel synthesis; however, they can be broadly categorized as either chemical crosslinking or physical crosslinking. Grafting, radical polymerization, click chemistry, enzymatic processes, thermo-gelation, and radiation crosslinking are all ways that can be used to covalently crosslink chemical hydrogels. In addition, polymers, such as alginate, that are abundant in anionic groups can be induced to gel by adding ions such as Ca^2+^, Mg^2+^, and Zn^2+^ to the hydrogel precursor. Both divalent and multivalent ions are used to induce gel formation. Hydrogels can be produced in the laboratory, but in nature, they are more likely to arise as the result of self-assembly via physical crosslinking processes, which encompass, primarily, the modification of intermolecular communication interactions within mechanisms such as ionic crosslinking, hydrophobic interactions, and hydrogen-bound gels. To achieve the appropriate hydrogel structure, however, several parameters can be regulated or modified during the gelation process [38,39]. Crosslinking can be either chemical or physical, and both methods can be used to create hydrogels. Hydrogels are typically made with hydrophilic monomers; however, hydrophobic monomers can be employed to achieve specific goals. Hydrophobic synthetic polymers are employed to give the hydrogels mechanical strength and longevity. Monomer, crosslinker, and initiator are the primary building blocks for hydrogels, with water serving as a diluent and a control for the reaction temperature. Hydrogels can undergo crosslinking reactions in several ways, including by the use of reactions and ionizing radiation to generate free radicals that recombine to form crosslinks, entanglements, electrostatic forces, and crystalline structures. Crosslinking processes join polymer chains to form networks in hydrogels, which are produced from polar monomers. Mechanical characteristics and viscoelasticity can be enhanced by such modifications, which have several potential pharmacological and biological uses [40]. The most common techniques for making physical and chemical gels are summarized in Figure 2.

### 3.1. Properties of Hydrogel

For the development of an effective gel delivery system, a fundamental familiarity with the gel characteristics is required. Studying the following characteristics will shed light on the gel–solute molecule interactions.

### 3.2. Swelling

Hydrogels are macromolecular polymeric networks that are crosslinked and can swell in a liquid. The expanded polymer functions as a filter, permitting only a certain amount of diffusion of the solute molecules. If the polymer network is crosslinked, it becomes insoluble and can hold the solvent by producing a gel. Because of the hydrophilic functional group linked to the backbone and the disparity in osmotic pressure between the gel phase and the solvent phase, hydrogels can absorb water. The rate of nutrient uptake and release in the hydrogel is dependent on the concentration of water within the gel. One subset of hydrogels with considerable medical promise is thermally sensitive materials. Although they have a liquid appearance at room temperature, when exposed to the body’s temperature, they transform into a viscous gel, which increases their staying time and slows their release rate. They can change volume or undergo a phase transition as the surrounding temperature fluctuates. Thermo selective gels, such as a water concentrate solution of poloxamer, are increasingly being employed in tissue engineering [41,42]. Furthermore, the release patterns of drugs and solvents from hydrogel polymeric networks are controlled by the rate and degree of swelling. Scientists employ a variety of techniques to calculate the percentages of free and bound water in comparison to the total water content. This result demonstrates the hydrogel’s ability to expand. The water content of hydrogels is often studied using tiny molecular probes, DSC, and proton NMR. The swelling qualities of a hydrogel can be evaluated to obtain insight into its mechanical capabilities, degree of crosslinking, rate of degradation, and other attributes. Crosslinked gels may be distinguished from the non-crosslinked parent polymer via the evaluation of the swelling and swollen state stability [43,44].

### 3.3. Mechanical Properties

A hydrogel’s mechanical properties are typically proportional to its water content and crosslinking density. A higher degree of crosslinking makes the gel stiffer, whereas heating softens the substance. A perfect hydrogel would have high rates of diffusion and responsiveness and be mechanically stable. Young’s modulus, Poisson’s modulus, the storage and loss moduli, etc., can be calculated to determine the hydrogel’s mechanical properties using a texture profile analyzer or rheometer. There has recently been a rise in the number of studies aimed at developing hydrogels with high mechanical performance. The fabrication of high-strength hydrogels has been accomplished by a variety of approaches, including double-network, topological, nanocomposite, macromolecular microsphere composite, and supramolecular hydrogels. These materials show promise as multifunctional scaffolds for tissue engineering due to their adequate and robust mechanical characteristics. Depending on where the hydrogel will be used, a certain stiffness level can be designed. A stiffer substance is needed, for instance, to seed osteoblast cells than is necessary for cultivating adipocytes [45]. However, characterizing materials and conducting tensile and compressive tests are fundamental techniques for gauging mechanical performance [46,47].

### 3.4. Crosslinking

Although crosslinking is not a fundamental property of hydrogels, it does have a significant impact on the other properties of the material. Because of this, the hydrogel is not only resistant to heat and erosion but also to mechanical stress. It has the potential to affect rheological parameters, skin hydration, and skin diffusion [48]. Although the nature of crosslinking can vary greatly, the degree of crosslinking is always related to every other property of a hydrogel. Physical crosslinking (by complicated coacervation or ionic contact), chemical crosslinking (by a crosslinker), and radiation crosslinking can all be used to generate the hydrogel network. The degree of crosslinking determines the material’s properties, allowing us to optimize it for a wide range of uses while still using a single polymer [49,50]. Nonetheless, there are a few drawbacks, such as their inflexibility in processing properties [51], because they are insoluble and infusible.

### 3.5. Polysaccharides

Polysaccharides are the biopolymer group with the longest and broadest experience in the biomedical area [52] thanks to their numerous benefits throughout the hydrogel manufacturing process. When compared to synthetic polymers, polysaccharides have several benefits due to the safety and biodegradability of the monomer residues and the fact that they are easily soluble in water. In addition, many polysaccharides are less expensive than synthetic polymers [53] because of how simple they are to produce. Hydrogels based on polysaccharides have recently been applied in drug delivery methods, as summarized in Table 1.

### 3.6. Chitosan

The hydrogel synthesis process frequently employs chitosan (CHI). Chitin removed from the exoskeletons of marine crustaceans is deacetylated to produce it. Chitin’s intra-chain hydrogen bonding gives it a strong crystalline structure and makes it relatively insoluble in water [60]. The percentage of glucosamine monomers in the chitin structure is proportional to the charge density of this polymer. Chitin is difficult to work with since it contains many acetylated groups. Chitin, which is insoluble in water containing acetic acid, is highly sensitive to this factor [61]. Chitin is transformed to CHI and becomes soluble in aqueous acidic environments when the degree of deacetylation is around 50%. The deacetylation of chitin to CHI increases the polymer’s amino group count, making it positively charged [62]. In addition, CHI demonstrates antibacterial activity due to the presence of amino groups in its backbone that facilitate binding to the negatively charged bacterial cell walls, hence modifying the cell envelope structures and permeability [63]. However, CHI’s crystalline structure and high molecular weight make it unsuitable for use in the food industry or the medical field. Hydrogels made from this biopolymer are unlike any others, and their cationic nature makes them ideal for use in drug administration [63,64].

### 3.7. Cellulose

**Figure 3:**
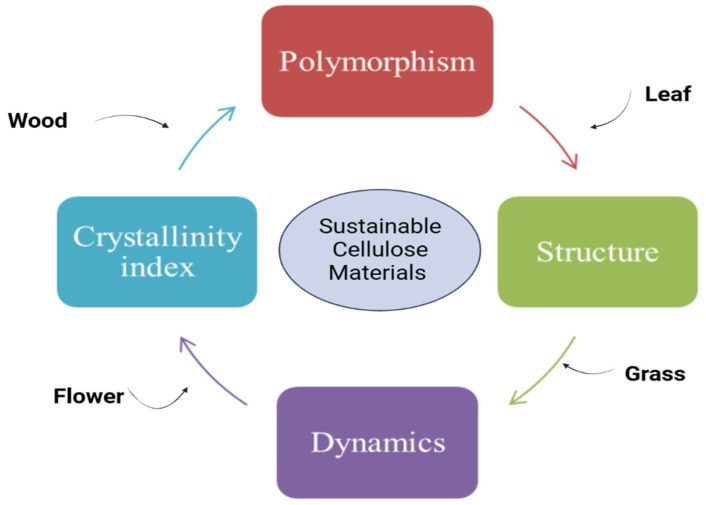
The ssNMR method is an advanced technology tool for characterizing sustainable cellulose-based products.

Cellulose hydrogels (CLS) are porous scaffolds with the potential to imitate the extracellular matrix in several ways [65,66]. These hydrogels have found applications in a wide range of biomedical fields, from wound dressings and bioimaging to targeted medication delivery and tissue engineering. Most natural fibers start with CLS since it is the most widespread biopolymer. It is biodegradable but still cheap, non-toxic, and mechanically and thermally stable [66]. Anhydro-D-glucose has a hydroxyl group on one end and a reducing functionality on the other, and it is held together by a -(1,4)-glycosidic bond [67]. Although CLS is insoluble in water, this problem can be remedied by several chemical modification processes including esterification, etherification, and oxidation. CMC has excellent stability across a broad pH range, from 3.5 to 10, and it has no flavor or odor [68,69].In recent decades, solid-state NMR has seen a radical transformation, evolving from a poor resolution, out-of-sight technology to a technique that is essential for determining the structure and dynamics of a wide variety of materials in a variety of physical states [70]. Since the active nuclear spins interact with the magnetic fields, solid NMR spectra are typically wide, with large line widths and low intensities [71]. Limited thermal movements and a lack of fast molecule tumbling give rise to orientation-dependent nuclear magnetic interactions in solid states. This slow motion reveals local geometric and electrical structures [72,73,74] in the form of several forms of internuclear and orientation-dependent nuclear interactions. Newly designed pulse sequences and other solid-state NMR techniques have been developed to reduce or remove the spectral breadth of solid materials [75]. The combination of solid-state NMR with the magic angle spinning (MAS) approach proved the most successful in suppressing anisotropic and dipolar interactions, which predominate in the solid state. As shown in [76], a sample is rapidly rotated at an angle of 54.47 degrees, concerning a stationary external magnetic field, along the vertical axis. The present implementation of the MAS technique into solid-state NMR, as shown in Figure 3, has the potential to improve resolution and the spectrum signal-to-noise ratio, thus providing critical chemical information at an ultrastructural level for cellulose-based materials under a variety of situations.

### 3.8. Characterization

The swelling, size, mechanical properties, and degradation rates of hydrogels can vary significantly depending on their purpose. Hydrogels have a viscous stiffness that is much lower than their elastic modulus, even in the plateau zone [75], although their elastic modulus has a definite plateau region that extends to times at least on the scale of seconds. Because of this combination of characteristics, hydrogels stand out among other types of viscoelastic polymers. The crosslink density determines the mechanical strength of these gels, but the more crosslinks there are, the less water they can hold. To characterize a hydrogel, one must first calculate its swelling capacity, a parameter with a range that is limited by the gel’s elastic forces. This implies that the hydrogel’s solubility is effectively boundless and that there are only limitations coming from the elastic stresses of the network. The ability of a hydrogel to absorb water is influenced by its porosity structure, the materials employed, and the crosslink density [76]. The swelling degree and elastic modulus of equilibrium hydrogels are additionally influenced by the crosslink and imposed polymer concentrations [77].

## 4. Modification of Sodium Alginate

### 4.1. Physical Blending Modification

Among the various techniques used to create novel polymeric composite materials, physical blending modification ranks among the simplest, most cost effective, and practically applicable. Sodium alginate’s abundance of hydroxyl and carboxyl groups found throughout its molecular structure facilitates the formation of intermolecular hydrogen bonds and, in turn, improves the mechanical characteristics of blended materials by interacting with the functional groups of other polymers [78]. Furthermore, these polymers’ characteristics may be preserved during mixing, allowing them to effectively make up for sodium alginate hydrogels’ shortcomings. The most popular way to enhance sodium alginate’s capabilities nowadays is via mixing with nanomaterials, natural biomaterials, and polymeric synthetics.

### 4.2. Synthetic Polymer Materials

Many synthetic polymers, such as polyvinyl alcohol (PVA), polyethylene oxide (PEO), poly(-caprolactone) (PCL), poly (lactic acid) (PLA), polyethylene glycol (PEG), etc., are commonly blended with sodium alginate for modifications, in addition to the natural biological materials mentioned above. Sodium alginate hydrogels can benefit greatly from the addition of these polymers because of their enhanced mechanical characteristics. When combined with sodium alginate, the pore structural features of synthetic polymers can be artificially adjusted from the nanoscale to the micrometer scale [79]. By increasing its viscosity [80], PVA not only makes sodium alginate solution easier to print but also increases the material’s porosity, which in turn facilitates protein adsorption and water/media permeability. However, when microporosity levels go too high, the resulting loss of mechanical characteristics [81] can be problematic. In contrast, increasing the tensile strength and elastic modulus of scaffolds can be achieved by incorporating magnesium oxide (MgO) into a PVA base. Since PEO is both biocompatible and non-toxic, it is frequently used with sodium alginate to boost the latter’s mechanical qualities [82]. This is because PEO’s ether oxygen may establish hydrogen bonds with sodium alginate’s hydroxyl groups. One of the most exciting areas of study in tissue engineering matrix materials currently is the creation of multifunctional hydrogels by combining the benefits of several materials. To boost their mechanical qualities, nanoparticles and polymers are frequently used as infiltrators. Composite hydrogels with excellent mechanical characteristics and electrical conductivity are commonly prepared by combining these two components with sodium alginate. Incorporating 1 wt% graphene nanosheet (Gr) hydrogels can result in an 18-fold reduction in the impedance of the composite scaffold, produce a broad conductive channel, and boost the toughness and strength by 4 and 3 times, respectively, due to Gr’s better electrical and mechanical properties. Cell attachment and spreading abilities were also boosted by 1.4 times, as shown by a PC12 proliferation assay. Sodium alginate hydrogels are commonly modified with gelatin, although the material’s weak mechanical qualities have been a problem for a long time. To boost hydrogels’ mechanical qualities, researchers have experimented with incorporating nanoparticles and polymeric components into SA/gelatin hybrid hydrogels.

### 4.3. Chemical Modification

Chemical modification is the process of creating a new substance by altering an existing one; in this case, sodium alginate is modified by adding a functional group to one of its hydroxyl or carboxyl groups [83]. Oxidation, sulfation, and grafting are common chemical techniques for modifying sodium alginate.

#### 4.3.1. Oxidation

Sodium alginate, generated by oxidizing the hydroxyl groups on glyoxal units with an oxidizing agent, such as sodium periodate, is one of the most widely used chemically modified forms of sodium alginate. Sodium alginate’s molecular weight can be lowered, and its rheological characteristics are improved by exposure to an oxidizing agent [83,84]. Sodium alginate is also highly challenging to eliminate from the body once it has been implanted, even though sodium alginate oxide (OA) compensates for the difference [53]. Increasing oxidation leads to increased solubility, decreased molecular weight, and a faster disintegration rate of Ca^2+^ crosslinked sodium alginate hydrogels [85,86,87,88]. Therefore, oxidized sodium alginate provides multiple benefits when making biodegradable scaffolding. However, the oxidation reaction is typically feasible at low concentrations of sodium alginate (4% or less) [84] because, as the oxidation degree increases, the elastic modulus of oxidized sodium alginate reduces, becoming lower than that of pure sodium alginate. The limitations in the use of oxidized SA are largely attributable to the material’s rapid degradability, which is harmful to the durability of scaffolds. Several investigators have looked into ways to strengthen oxidized sodium alginate’s flimsy mechanical characteristics. Sodium alginate oxide’s mechanical characteristics can be enhanced with the help of crosslinking agents. During the oxidation of sodium alginate, the number of aldehyde groups increases, and these groups can undergo Schiff’s base reaction with amino/hydrazide-modified polymers to generate covalent crosslinks. This crosslinking method can be used to create structurally stable composite hydrogels without the use of an exogenous crosslinking agent or any other crosslinking process [89,90]. Sodium alginate oxide, polyethene glycol, and chitosan can be used together to make an injectable hydrogel that self-crosslinks. The composite hydrogel improves degradation rates and has advantageous rheology and non-toxic properties. Sodium alginate oxide’s mechanical characteristics are improved by PEG’s ability to establish physical entanglement, leading to a higher compressive strength (126 kPa) [91]. The cytocompatibility and electrical conductivity of the hydrogel made from a combination of OA, pyrrole (py), and gelatin are both high. Because pyrrole penetration lowers the hybrid hydrogel’s viscosity, it can be easily extruded and used in 3D printing. However, the oxidation of pyrrole produces polypyrrole, which improves the hybrid hydrogel’s rigidity and electrical conductivity and strengthens the scaffold’s structural integrity [92]. The limitations of oxidized sodium alginate’s mechanical qualities can be mitigated by blending it with other polymers, thus expanding its use in tissue engineering. Oxidized sodium alginate’s limiting mechanical qualities can be mitigated by blending it with other polymers, opening new possibilities for its use in tissue engineering.

#### 4.3.2. Sulfation

A substantial amount of sodium alginate sulfation occurs at the molecular level via sulfate molecules. Sulfating sodium alginate leads it to break down hydrogen bonds because the hydroxyl molecules are substituted by sulfate groups. Sulfated sodium alginate is a sulfated glycosaminoglycan (sGAG)-simulating molecule with applications in cartilage, nerves, and other tissues. Cell proliferation, migration, and differentiation all have been influenced by a variety of growth factors, some of which are bound by sGAG in tissues and organs [93,94,95]. Sodium alginate sulfate possesses a strong affinity for an extensive range of heparins since its negative charge is attracted to the positively charged groups of amino acids found in proteins. Sulfated sodium alginate has additionally been shown to stimulate cell growth by binding to and activating various growth factors. Vascular endothelial growth factor, tumor growth factor, hepatocyte growth factor, insulin-like growth factor (IGF), and basic fibroblast growth factor are all examples of growth factors. Via these growth factors, sulfated sodium alginate stimulates the growth of new coronary blood arteries due to its anticoagulant and hemocompatibility qualities [96,97,98].

#### 4.3.3. Graft Copolymerization

Sodium alginate with a carbodiimide carboxy coupling to include peptide side chains of sodium alginate is another modification technique that has garnered considerable interest. RGD-modified sodium alginate is preferred because it is simple to attach to the sodium alginate framework using water-soluble carbodiimide, as well as because the resulting altered sodium alginate has been successful at promoting cell attachment, survival, and proliferation by enhancing the expression of vascular growth factors [99,100,101,102,103]. Significantly improving the mechanical characteristics of RGD-modified sodium alginate scaffolds will require further research. Hydrogels with highly tunable stress relaxation can be made by blending PEG with sodium alginate physically, and PEG can also be grafted onto the molecular chains of sodium alginate. By modifying the PEG molecular weight and number, the elastic modulus of PEG-SA hydrogels can be modified. Hydrogels made from PEG-SA can have faster stress relaxation times if more PEG chains are used in their construction, which in turn can facilitate cell proliferation and diffusion. However, RGD-coupled PEGSA hydrogels specifically integrate the beneficial effects of RGD grafting and PEG grafting to improve the growth and reproduction of fibroblasts while additionally improving the differentiation of mesenchymal stem cells [104]. Future studies on peptides as a functionalizing agent for sodium alginate, as well as the development of novel forms of sodium alginate with modifiable chemical and physical properties, are expected. When calcium ions are not utilized to crosslink sodium alginate sufficiently quickly, the scaffold immediately loses its mechanical integrity and could potentially collapse [105]. This is because multivalent cations are crucial to the mechanical stability of sodium alginate scaffolds. Scaffolds’ mechanical characteristics and stability can be enhanced via the chemical manipulation of the intermolecular crosslinking mechanism.

### 4.4. Double-Network SA Hydrogels

A Double-Network hydrogel (DN) was initially proposed by Gong [106] of Japan’s Hokkaido University. Dual-network hydrogels have a neutral network structure that is employed to bridge the internal gap of the electrolyte network structure, which has a high crosslinking density but low mechanical properties [107]. Hydrogels’ strength and mechanical capabilities have been enhanced by the dual-network system without any discernible loss of the material’s original properties. This was possible owing partially to the internal breakdown of covalent connections in the first network, which absorbed energy and improved the resistance of cracks. The Gong group also conceptualized and demonstrated this “sacrificial bond” mechanism. A wide variety of crosslinking techniques (physical crosslinking, chemical crosslinking, physical radiation crosslinking, and many more) have been utilized in the production of DN hydrogels. There are currently ten million different varieties of SA hydrogels thanks to extensive study. Materials are reviewed based on the areas of study that have received the most attention in recent years; these include polyvinyl alcohol (PVA), alginate (Alg), and protein for use in the preparation of basal hydrogels to improve their mechanical properties [108].

#### 4.4.1. Polyvinyl Alcohol Hydrogels

Polyvinyl alcohol (PVA) is a polyhydroxy polymer that may be dissolved in water. Because of its safety for humans and the environment as well as its biodegradability, it finds extensive application in the fields of medicine and biology. Due to the high concentration of hydroxyl groups on PVA molecules, hydrogels can be created by both chemical or physical crosslinking. The PVA hydrogel microcrystals created by physical crosslinking forma three-dimensional network and are essential for improving mechanical performance. While freezing, the hydrogel’s internal network becomes more compact, and relative crystallinity increases. PVA hydrogel’s strength was found to change with both the length of time it was frozen and the number of times it was frozen and thawed [109]. The mechanical strength of single PVA hydrogel is still not up to the desired impact in some applications [110,111]. As a result, taking PVA as the first network and introducing the second network material to make double-network hydrogel has become a research hotspot. High-strength PVA hydrogel was created by adding tannic acid in dimethyl sulfoxide and water. Hydrogen bonds formed between the PVA molecule’s hydroxyl group and the tannic acid’s phenolic hydroxyl group, hence controlling the concentration of tannic acid. The hydrogel’s mechanical strength increased and subsequently decreased over time. The maximum tensile strength of the hydrogel was measured at 2.12 MPa. This means that a 30% TA concentration in normal saline can boost the mechanical strength of a PVA/TA hydrogel by 654%, allowing for a maximum tensile strength of 16 MPa. This is because typical saltwater serves as a “sacrifice domain” in the hydrogel structure, absorbing the energy created when hydrogen bonds are formed between molecules. The hydrogel is quite strong under tension [112].

#### 4.4.2. Alginate Hydrogel

Alginate (Alg) is a natural polymer made up of b-1, 4-D-mannan acid (M) and a-1, 4-L-glucuronic acid (G), and it finds many applications in the medical and food industries due to its high biocompatibility. Ca^2+^ is effective in dissolving alginate into the hydrogel, and Ca^2+^ readily combines with other 2-valent cations in the G region, providing a solution to the issue of hydrogels’ weak mechanical characteristics. The complete crosslinking of PAM (polyacrylamide) before the ion exchange of Alg will result in poor solvent exchange, and the two polymers will aggregate on a micron scale. A massive crosslinking zone forms after water absorption as the freshly produced alginate aggregates are stabilized by hydrogen bonding and metal–ligand interaction. It is beneficial for strengthening the mechanical properties of hydrogels [113]. As reported by Zhao [114], the hydrogen bond interaction in SA/PAM (polyacrylamide/sodium alginate) hydrogel enabled SA self-assembly in the porous matrix of PAM, and the layered semi-interpenetrating network structure improved the hydrogel’s mechanical qualities. Zhang [115] employed radiation technology and Cu^2+^ crosslinking to create a polyacrylamide/copper alginate (PAM/Cu-Alg) double-network hydrogel, which he then used to analyze the effects of varying alginate contents, Cu^2+^ concentrations, and absorbed irradiation doses. The inclusion of Cu^2+^ will enhance the hydrogel’s conductivity, and the degree to which Alg crosslinks with Cu^2+^ will have a major impact on the hydrogel’s mechanical properties. This is because a great deal of energy is lost due to the interactions of ions between Alg and Cu^2+^ and the hydrogen bond between PAM chains. The compression modulus, strength, failure strain, and toughness of the hydrogel all increased with an SA concentration from 0% to 2% compared to their measurements taken before SA was incorporated into polyethylene glycol (PEG) (meth). As a result, the hydrogel’s performance was enhanced thanks to the SA network ability to link and the PEG (meth) acrylate covalent network.

#### 4.4.3. Protein-Based Hydrogels

Hydrogels made of polymers can be classified as either natural or synthetic. Both PVA and SA, as well as the majority of modern hydrogels, are man-made hydrogels. The development of synthetic polymer hydrogel is hampered by the material’s biodegradability and probable toxicity, despite the material’s promising mechanical properties and robust swelling capability. Hydrogels made from natural polymers, such as proteins, have been the subject of methodological and practical proposals from scientists in recent years. When compared to synthetic macromolecules, proteins’ biocompatibility and biodegradability are far superior. Researchers have taken notice of protein-based hydrogels because of their potential utility in a variety of biomedical settings. The poor mechanical capabilities of pure protein hydrogels result from their otherwise advantageous biocompatibility and homogeneous structure [116,117], as well as the limits imposed by the single protein network.

## 5. Characterization of Sodium-Alginate-Based Hydrogels

Alginates are derived from brown seaweeds (Phaeophyceae), in which the polysaccharides play a crucial role in the thalli’s structural integrity. The alginate polysaccharide is linear in shape and composed of alternating blocks of homopolymeric (MM or GG) and heteropolymeric (MG) 1,4-linked -d-mannuronic acid (M) and -l-guluronic acid (G) units. The amount of M- and G-units and the block structure of seaweed are determined by its species, geographic location, season, vegetative phase, and the collected fraction of the algae species. Mannuronan C-5 epimerase [118] is responsible for controlling the M/G ratio in algae. Alginate hydrocolloids’ gelling qualities are based on the distribution of M- and G-units, as well as the counter ions present, whereas the hydrocolloids’ viscosity is dictated by their average molecular weight. Hard, inflexible gels are typical of those with a low M/G-ratio, whereas soft, malleable gels are typical of those with a high M/G-ratio. Brown seaweed and bacteria are both viable sources for the hydrogel polymer sodium alginate (SA). It is used in tissue engineering and for the targeted distribution of proteins and medicines [119,120] due to its biodegradability and high biocompatibility. Alginate is now known to be a type of linear copolymer in which M and G blocks are connected by 1,4-d-mannuronate and 1,4-l-guluronate residues, respectively. Gels made from l-guluronate in alginic acid are strong but brittle, while gels formed from d-mannuronate in alginic acid are weaker but more flexible [121]. The heteropolymer structure of alginic acid is linear. Because of their high G-content, low M: G alginates have found widespread use in a variety of fields, including environmental remediation, biomedicine, pharmaceuticals, food additives, and industry [122]. Medical applications such as drug administration and regenerative therapy benefit greatly from hydrogels’ capacity to (over time) breakdown into physiological metabolites under specific conditions. Hydrogels that mimic natural extracellular matrices and cell adhesion surfaces are useful for such applications because they facilitate the deployment of cells and their subsequent proliferation [123]. MG heteropolymeric blocks are interspersed with M and G homopolymeric blocks in naturally occurring alginates, which are linear polysaccharide chains. The food industry was not the only one to benefit from industrial alginate manufacturing; the industrial and medicinal sectors benefited as well [124]. The pharmaceutical industry uses this for a wide variety of purposes, including cancer treatment, protein, and cell delivery, and oral or controlled-release delivery [125]. Water content is intimately related to various hydrogel characteristics. The hydrogel is mostly water, and this water can be further broken down into two groups: waters that are highly associated and waters that are weakly related [126]. This categorization is based on the strength of the hydrogen bonds between water molecules and the alginate matrix. Some water molecules contact the hydrophilic groups in alginate for long periods, while other waters reside in the macropores, where they can move freely and only weakly interact with the polymers. In addition to providing information on the presence and size of the macropores, the latter macropore waters interact with the enclosed payload. Therefore, finding and researching these seas is crucial. Water molecules in hydrogels that are restricted and involved in strong hydrogen bonding to the polymer are resistant to freezing below 0°, making this a useful criterion for classifying (and detecting) different types of hydrogel waters. Other (biological) settings also exhibit this tendency [127,128]. The water in hydrogels can be divided into three categories: (1) water that does not freeze (highly bonded alginate), (2) water that has a freezing point like bulk water, and (3) water that freezes at a lower temperature inside the hydrogel [129,130,131]. Alternatively, hydrogel waters can be classified based on their mobility: water that is immobile, owing to strong alginate binding, is called “bound” water; water that is dynamic due to the absence of binding is called “free” water; and water that exhibits intermediate mobility is called “transient” water. Water is said to be “free” when it can move through or around the matrix (tissue) with minimal resistance and without interacting much with the alginate [132,133]. Bound water is water that is firmly attached to the matrix (in this case, alginate) and cannot move or freeze. Entrapped water is water that is encased by the structural features of the matrix but has weaker or temporary interactions with the matrix, giving it intermediate mobility. The mobility of these water molecules is restricted (in comparison to “free” water) [134]. Depending on the proximity and diffusion rates of the water pools, there may be an interchange between different pools. One advantageous element of this is that the relative amounts of these various hydrogel fluids reflect structurally and functionally significant characteristics such as the hydrogel mesh size and macropore size, with macropores filled with encapsulated but not tightly linked water molecules.

### 5.1. NMR Spectroscopy

NMR spectroscopy is well known for its application in liquid or solution states. Rapid thermal isotropic motions experienced by tiny soluble molecules in the solution state average out all orientation-dependent nuclear magnetic interactions. The resulting NMR spectra from a solution have a high signal-to-noise ratio because only isotropic components interact with it. Molecules in a “solid” state cause issues because they cannot tumble rapidly due to their size and restricted motions. Since “solid-state” NMR experiments do not involve small, dissolved molecules, the presence of orientation-dependent nuclear and internuclear interactions (such as anisotropic and dipolar interactions) is revealed. However, the resolution loss, decreased sensitivity, and difficulty in detecting individual atomic sites due to line broadening are all costs associated with these interactions, which provide insight into the local geometric and electronic structure [76]. The NMR spectra of most materials are broad and weak without line-narrowing procedures, which severely restrict the amount of information that can be gleaned from this method. Several methods have been created, however, to recover sharpness and sensitivity. Magic angle spinning (MAS) is frequently used in conjunction with solid-state NMR to dampen the dominant anisotropic interactions in the solid state. As part of this method, the sample is rapidly rotated at an angle of 54.74 degrees relative to the NMR instrument’s static magnetic field. If the MAS frequency is higher than the amplitude of the interaction, then the unwanted line-broadening interaction will be completely suppressed. As a result, the isotropic chemical shift frequencies observed in liquid-state NMR spectroscopy are shown to occur at the same frequencies in the solid-state NMR spectrum. The increasing speed of MAS has vastly improved the capabilities of current solid-state NMR. Since solid-state NMR spectroscopy using MAS-based techniques can provide in-depth molecular information without causing any damage or harm, it has become widely used in the pharmaceutical and biomedical industries [135,136,137,138,139]. MAS NMR provides structural and molecular dynamical information in a variety of non-crystalline environments, including amorphous and gel-like ones, in which other typical solid-state techniques fall short. Table 2 summarizes some important distinctions between solid- and liquid-state NMR.

### 5.2. Advantages of NMR Spectroscopy

The development of nuclear magnetic resonance (NMR) spectroscopy has been one of the most important contributions to the field of analytical science in recent decades. NMR has been used to study everything from a single cell to entire organs and tissues in both the biological and nonbiological sectors. Strong and consistent magnetic fields are needed for NMR. The magnitude of a magnet’s pull is expressed in tesla or megahertz. For NMR to work, the magnetic field strength must be represented by a reference nucleus. However, there is a risk of overexposure to radiation associated with the widespread use of electromagnetic spectra in healthcare and dentistry for the detection of abnormalities, fractures, and the monitoring of healing tissues. However, prolonged exposure to X-ray radiation can have negative consequences, such as cellular damage, even though it is painless and quick. In recent years, a plethora of cutting-edge analytical technologies that can provide pinpoint results with minimal tissue injury has emerged. In the 1940s, scientists made the initial discovery of nuclear magnetic resonance (NMR) [141].

### 5.3. Surface-Enhanced Raman Spectroscopy

Alginic acid is the major structural polysaccharide present in all brown seaweeds (Phaeophyta); it is a linear copolymer of *β*-D-mannopyranuronic acid (M) and *α*-L-gulopyranuronic acid (L) linked 1→4, which are arranged in homopolymeric and heteropolymeric blocks. There was no link found between M/G ratios and block composition in alginates, and the proportion of uronic acids in each species or tissue type varied widely. The characterization of alginic acid samples and block fractions using vibrational spectroscopy revealed that the FT-IR spectra of the homopolymannuronic and homopolyguluronic acid fractions displayed distinctive bands [142]. Raman spectroscopy has been used to identify alginates, as described by Pereira et al. [143], and IR, Raman, and NIR spectroscopies and chemometrics have been used to determine the M/G ratio in alginic acid, as reported by Salomonsen et al. [144]. Alginic acid salts have been reported as model compounds for use in the Raman spectroscopy study of biofilm matrix [145]. However, the high fluorescence of biological systems can mask the vibrational signals, and the low concentration of the samples results in poor spectra, limiting the utility of Raman and IR spectroscopies. Vibrational spectroscopy, which is enhanced by metal surfaces, has the potential to address these limitations since it may be used with low concentrations of analytes and because the effect of metal nanoparticles suppresses the inherent fluorescence of the materials [146]. In addition, measurements can be performed in aqueous conditions when metal colloids are used, which may facilitate conformational investigations of these macromolecules. The analytical method of surface-enhanced Raman spectroscopy (SERS) is particularly useful for elucidating the molecular structures of complicated substances [147]. Raman signals of molecules can be amplified by the electromagnetic field surrounding all the nanoparticles, which may increase the total vibrational signal by as much as 106 times. The first stage in Raman amplification was accomplished by Fleischmann et al. [147] using an electrochemical method with surface-adsorbed molecules of pyridine in a silver electrode. The detection of proteins, amino acids, peptides, and other biomolecules at concentrations as low as 1012 to 1014 M using SERS has now been reported in many publications [148]. Schmid et al. [149] recently examined alginate samples containing Ag colloids by tip-enhanced Raman spectroscopy.

### 5.4. Methodology of Sodium Alginate Hydrogel

Hydrogels made from alginate are just one example of biomaterial engineering that has benefited from the ever-expanding field of material science. The internal and diffusive gelling that occurs during construction is vital to many aspects of the final product. Gelation proceeds in two sequential reagents, with calcium ions becoming increasingly prominent within the body of the alginate, after a calculated injection of the calcium chloride solution into the alginate barrage. Numerous critical factors depend on the internal and diffusive gelling that occurs during building. Scaffolding makes it hard to maintain control [150]. The second approach involves using a double-nozzle procedure attached via a triad method of stopcock to manipulate the calcium chloride solutions and the alginate until the desired pliable hydrogel is achieved [151]. The technique’s main strength is how simple it is to generate a gel-like state consistently. Having a mechanical duty is just one of the many reasons why institutions can be utilized to make sodium alginate hydrogel scaffolds. To facilitate the process of alginate hydrogel injection, it is common to practice combining the alginate and calcium ions mechanically, as in the case of using a homogenization technique to combine the calcium gluconate solution and the marine alginate. Another technique involves subjecting the alginate solution to a barrage of divalent ions (a calcium chloride solution, for instance), which then facilitates the diffusion of calcium ions into the material’s core during the gelation process, beginning on the alginate’s outer surface. Using this method, one can create tissue scaffolds with a complicated architecture by manipulating alginate in a variety of fabrication procedures. However, bio-fabrication processes rely heavily on the presence of biomolecules and living cells, and the concentration of calcium ions is seen as an important goal for both [151,152]. Customized hydrogel materials also have various applications outside chemical catalysis, including nano-engineering, nanomedicines, nano-energy, and the visualization of reactions in aqueous media.

## 6. In Vitro Models of Development

Scaffolds have been the primary focus of tissue engineering research for tissue regeneration in transplantation therapies; however, other potential applications for this field, including drug efficacy and toxicology studies, as well as morphogenesis and tissue development studies, are now being investigated. A significant cost and regulatory input are provided by an in vitro tissue model throughout in vivo model creation and execution [153], enabling investigators to make advances in the mechanistic examination of simple, regulated systems. Conventional 2D culture systems show cells adhering directly to the substrate (cell-to-cell interactions) and then migrating away from the tissues [154,155]. Therefore, cells and tissues often exhibit quite distinct behaviors and experience significant variations in gene expression when cultivated in a non-natural 2D environment as opposed to a natural 3D environment [156,157,158,159,160,161,162]. Embryos may be maintained in a 3D culture system, while tissue architecture can be optimized for simple physical contact with the surrounding environment, and both of these are very representative of in vivo development.

## 7. Applications

**Figure 4:**
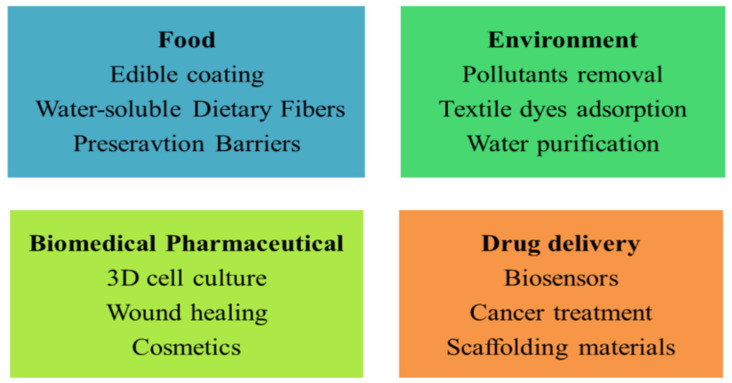
Schematics of alginate applications.

It has been established that alginate is biocompatible and non-toxic, making it ideal for usage in the food sector as an intensifier for a variety of applications, including salad dressings [163]. Alginate gels are non-invasive and can be injected orally for administration [34]. They can also be altered to increase the controlled release of macromolecules. Due to its superfine gelation technique, low cost, and recent relocation, research on alginate has recently shifted toward tissue engineering applications [163,164,165]. Alginate hydrogels are frequently employed in cell transplantation since they are biodegradable and simple to interact with [165]. In addition to neural stem cells, articular chondrocytes, skeletal myoblasts, and mouse embryonic stem cells in alginate gels may be employed to culture several distinct kinds of cells. The use of 3D scaffolds as part of a tissue engineering strategy for cell and tissue culture is becoming recognized as the most effective way to generate results in in vitro culture models that are predictive of in vivo conditions. A polysaccharide generated by brown algae with a linear structure, called alginate, has various desirable properties that make it a good candidate for use as an extracellular 3D matrix in tissue models and in vitro cell cultures. Hydrogels can encase tissues and cells extensively when exposed to a divalent cation because they can create intimate crosslinkages with the ions in their vicinity [166,167]. The alginate hydrogel can be biologically changed to facilitate specific interactions between adjacent cells or tissues, or it can be left undisturbed to provide mechanical support for tissue morphogenesis and/or biological reactions. Investigators have employed alginate hydrogel to develop a double system of cultural separation that interacts as a targeted approach to generate operational and developmental in vitro models [168]. Figure 4 provides an overview of the applications for materials based on alginate.

### 7.1. Nanomaterials

Nanomaterials that are multifunctional and extremely porous and that have a high surface-to-volume ratio [93,94] can create highly linked nanofibrous networks for sodium alginate. This means that sodium alginate can benefit from nanocomposite improvement. There are several ways in which the scaffold could benefit from being blended, such as (1) enhanced adherence of cells, proliferation, and distinction within the scaffold; (2) modified mechanical properties; (3) enhanced shear thinning capabilities of the bioink; and (4) enhanced biological and conductive properties of the scaffold [169,170,171,172]. Scaffolds constructed with composites containing 1% *w*/*v* single-walled carbon nanotubes combined with sodium alginate as the reinforcement had a 23.3% boost in tensile strength, a 3.3% boost in primary modulus, and a 49.7% boost in secondary modulus, compared to their unreinforced equivalents. After co-culturing with different cell types, rat cardiac endothelial cells showed substantial improvements in adhesion and proliferation [173,174]. Nanocellulose (CNCs, CNFs, CNCTs, and CNFTs) is added to hydrogel to boost the pore size, which subsequently enhances the material’s mechanical properties and stability [26]. This mixture aids in nutrition transfer and cell growth. CNCs are an ideal element that may increase the bioink’s shear thinning properties [175,176], which allows the better printing of sodium-alginate-based hydrogels. The high-precision printing of complex 3D structural scaffolds that resist degradation for up to 30 days [177,178] is made feasible by combining CNFs with sodium alginate. CNFs can improve bioink printing by eliminating waste, increasing bioink tolerance to physiological deformations, and permitting the good shape accuracy of molded scaffolds. Incorporating carbon nanotubes and nanocellulose into sodium alginate hydrogels, as well as the nanoparticles of hydroxyapatite (HAP) and mesoporous silica (MSN), may enhance their characteristics [179,180,181,182,183,184]. These components enhance the mechanical characteristics of the hydrogels as well as promote cell proliferation and differentiation, both of which are critical for successful cartilage tissue engineering. Therefore, the nanoparticle co-modification of sodium alginate hydrogel can greatly enhance both the physicochemical properties and the bioactivity of the material [185,186,187]. However, nanoparticles and sodium alginate have a powerful intermolecular interaction that frequently decreases the porosity of sodium-alginate-based hydrogels and makes degradation more challenging as the microporous structure is lost. Furthermore, nanoparticle aggregation has proven to be a difficult issue [188,189,190,191].

### 7.2. Tissue Engineering

**Figure 5:**
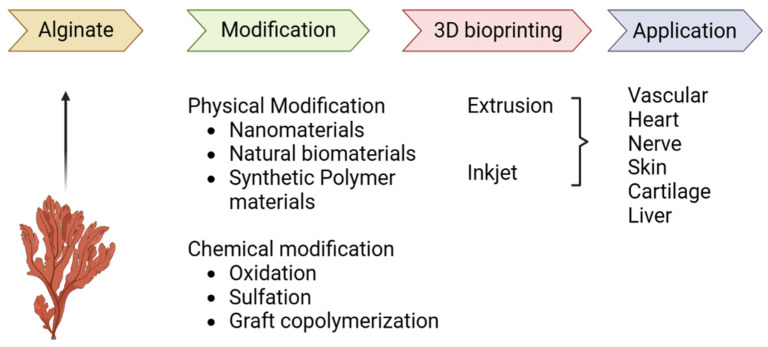
Schematic of sodium alginate hydrogel modification, 3D bioprinting, and application.

There are significant constraints to the standard transplantation techniques that depend on autologous or allogeneic grafts, which include the absence of donor tissues and severe immune reactions [192]. Tissue engineering can revolutionize healthcare by making it possible for the effective replacement of dysfunctional organs and tissues with artificial ones [193]. Tissue engineering is the implementation of engineering concepts in the study of living organisms to generate functional tissue substitutes. The use of autologous or allogeneic cells and the ability to modify in vitro culture conditions to mimic those that occur in vivo [194] are just two of the many advantages of tissue engineering. There are three essential components necessary for the basic idea of tissue engineering to work [167]. In the first place, live cells are required to generate the planned tissue. Donor tissue or stem/progenitor cells are common sources of the cells [193,194,195,196,197]. Second, a 3D scaffold is required to sustain cell-to-cell communication and provide structural support for developing tissue. By mimicking the tissue’s mechanical attributes and chemical signals, such scaffolds successfully recreate in vivo settings. Scaffolds used in tissue engineering can be constructed from a wide range of materials [198]. Some examples of synthetic scaffolding substances are polyglycolic and polylactic polymers, whereas examples of natural scaffold materials include collagen, alginate, and chitosan. Tissue engineering biomatrix materials and molding procedures are acknowledged to be the primary determinants of biomimetic manufacturing depth. Sodium-alginate-based hydrogels are used in a wide range of applications, and this inquiry aimed to provide a complete description of the current state of the modification research, molding process, and utilization level [199]. The contributors summarized the different ways sodium alginate can be modified, as well as the benefits and drawbacks of these altered hydrogels. Soft tissue engineering applications of sodium-alginate-based hydrogels have been analyzed along with the challenges and promise of using a 3D printing technique to manufacture such hydrogels, and a potential future route for research was expected. Figure 5 provides an overview of 3D bioprinting, modified sodium alginate hydrogels, and their potential applications. Tissue engineering (TE) is a multidisciplinary area that combines life science and materials science to stimulate tissue regeneration and repair [200,201]. Tissue-specific cells, scaffold materials, and cellular phenotype-guiding signals are the three main prerequisites for TE [202]. Scaffolds are essential to the TE method. Scaffolds provide a short-term structural base for damaged tissues to adhere, develop, divide, and regenerate. Hydrogels are an important class of scaffold materials. Hydrogels are polymer networks that are hydrophilic and may large amounts of water or biological fluids. Hydrogels have attracted interest for their TE applications as injectable scaffolds, which can be used to deliver cells into the body with minimal invasion, and for the development of matrices for cell encapsulation and for biofabricating 3D scaffolds. Because of their biocompatibility and biodegradability, hydrogels based on natural polymers are excellent scaffolds for TE. Cell attachment, development, and differentiation may be promoted in their natural form. Because they resemble the extracellular matrix of human tissues, hydrogels based on alginate have found extensive use in TE [203,204]. Linear polysaccharides called alginates are produced by both algae and bacteria and have an anionic charge.

### 7.3. The Implementation of Sodium Alginate Hydrogel in Regenerative Medicine

Regenerative and reparative tissue engineering relies heavily on the use of 3D scaffolds because, in comparison to a 2D culture, it better promotes cell adhesion and proliferation. Simulating the in vivo milieu and laying the groundwork for transplantation experiments are both made easier with the help of 3D-printed constructs that look and function like human tissues [203]. Tissue engineering applications for sodium-alginate-based hydrogels are being significantly enhanced using 3D printing technology. Different types of soft tissue, including the arteries, veins, heart muscle, epidermis, liver, and cartilage, have profited from the utilization of sodium alginate hydrogels for regeneration.

### 7.4. Skin

The skin is the largest body part and the body’s initial layer of protection against harmful elements. For quicker recovery from skin wounds, burns, and cuts, the clinical regeneration of the skin is one of the most prospective treatments in medicine, even though it would be necessary to print artificial skin tissue with biological functions and physiological properties [204]. Human skin consists of multiple layers of tissue, including the subcutaneous layer, the epidermis, and the dermis. Bioprinted skin requires it to be biocompatible, have mechanical characteristics compatible with skin tissues, and feature a well-linked capillary network [204] to facilitate the efficient exchange of nutrients and optimal skin regeneration. The hydrophilicity of sodium-alginate-based hydrogel helps to generate a moist wound environment, which speeds up the healing process for skin wounds. Additionally, the changed hydrogel’s mechanical properties are indistinguishable from those of healthy skin tissue [205,206,207,208,209]. Homogeneous porous scaffolds can be developed thanks to advancements in 3D bioprinting technology; a pore size of 100–300 m is optimal for cell activity and nutrition transfer [210]. The most popular biomaterials for use as scaffolds are SA/gelatin mixtures.

### 7.5. Vascular

The vascular system encourages the body in distributing oxygen and nutrients via diffusion, transferring blood, and eliminating waste. Neovascularization is essential for wound healing and organ remodeling [211], and it has been associated with the effectiveness of transplanting cells into scaffolds. Cell survival and function are dependent on vascularization in tissue engineering [212], and angiogenesis is required for soft tissue regeneration and recovery in organs such as the heart, nerves, muscles, and skin [213]. Some ischemic illnesses (myocardial infarction, hind limb ischemia, etc.) are effectively treated, in part, by stimulating vascular regeneration [214]. Bioprinting allows for the creation of three-dimensional scaffolds that promote vascular development and angiogenesis. Sodium alginate is the finest option if you need a scaffolding material. Vascular tissue regeneration makes extensive use of sodium alginate because of its ability to promote angiogenesis by (i) supplying growth factors to scaffolds to stimulate vascular growth; (ii) encapsulating different cells to enhance vascular growth; and (iii) preparing artificial tubular structures with microchannels to mimic blood vessels and deliver blood, nutrients, oxygen, etc.

### 7.6. Muscle

Muscle is the most abundant tissue in the body (accounting for about 45 percent of total body mass) and is primarily found close to bone [215]. Because muscles are innervated by peripheral nerves, the atrophy of the innervated muscles occurs when peripheral nerves are injured. This is especially true in the motor organs. Muscle also has many blood vessels; therefore, regenerating muscles with hollow tube networks can help with issues including poor nutrient delivery, oxygen imbalances, and waste elimination during the repair process [216]. Therefore, muscle regeneration can benefit from both nerve regeneration and vascular regeneration techniques. The administration of growth factors and the transplantation of cells are the two main methods now employed for muscle regeneration. Among these, sodium alginate and, in particular, cell-containing sodium-alginate-based biomaterial are the most widely utilized for 3D printing skeletal muscle. Sodium alginate has been shown to improve muscle regeneration by delivering growth factors (VEGF and PDGF) [217], with VEGF promoting angiogenesis in the limb and IGF-1 promoting muscle fiber regeneration and preventing cell death [218].

### 7.7. Heart

The current leading cause of death globally is cardiovascular disease (31% of total deaths). Atherosclerosis, which is defined by the accumulation of plaque contributing to the narrowing of arteries and tiny blood vessels, is mostly to blame for this. Myocardial infarction congestive heart failure, stroke, and valvular heart disease are just a few of the cardiovascular issues covered on the DVDs. Approximately 70% of these instances are attributed to myocardial infarction (MI), whereby the primary issue is the incapacity of impaired myocardial cells to undergo self-repair and regeneration [219,220]. The application of regenerative medicine in conjunction with tissue engineering is a significant factor in the restoration of damaged or diseased organs, particularly in the context of cardiovascular diseases and cardiac repair. Biomaterials intended for use in cardiac tissue engineering must share key properties with the native cardiac extracellular matrix and be able to support the microenvironment of host cardiomyocytes. Some of these features are the ability to conduct electricity, to be mechanically stable, to be elastic, to be bioactive, and to enable vascularization [212]. Sodium alginate has recently gained attention as a potentially game-changing biomaterial for use in heart recellularization, vascularization, and regeneration. It has undergone extensive clinical trials and is widely regarded as one of the most thoroughly tested biomaterials in this field [221]. Its primary applications are as follows:

- The restoration of ischemic myocardium can be assisted by chemicals and cells being transported, cells generating growth factors in a particular location, and new blood vessels being generated by cells, as observed by multiple researchers [222,223]. Sodium alginate, which has been sulfated and possesses a structure similar to that of heparin, can administer diverse growth factors and encourage myocardial angiogenesis, as evidenced by the results of [222]. Myocardial stress and apoptosis become successfully reduced, and unfavorable LV remodeling is limited, employing post-infarction mechanical characteristics and biological signals as design criteria [224,225,226] to inform material development. Sodium alginate and fullerenol are two examples of nanomaterial hydrogels that are employed to reduce cardiac stress and give long-term physiological and mechanical support to damaged heart tissue.
- Heart rate stabilization and cardiac contractility restoration following infarction with the administration of electrical impulses [219]. Myocardial infarction causes changes in behavior such as interruption of the normal heart conduction system due to damage to ion channels and connexins. Hydrogels made from sodium alginate that carry electricity could be used to restore heart contractility by delivering electrical impulses and keeping the heartbeat steady [219]. The regeneration of myocardial vasculature is a crucial aspect of tissue engineering for cardiac tissue regeneration. Prior research has employed hydrogel injection to promote cellular proliferation and differentiation, thereby facilitating the generation of blood vessels [227,228,229].

### 7.8. Alginate Hydrogel in Biomedical Applications

Alginate plays a significant role in the field of pharmaceutics by serving as a versatile agent with various functions such as thickening, stabilizing, and gel forming. Its ability to regulate the release of drug products is particularly noteworthy. Water-soluble, biodegradable, non-toxic, and nonirritant alginates are a type of naturally occurring colloidal polysaccharide. These are typically derived from several brown marine algae species [230]. Alginate is widely used in pharmaceutical applications, particularly in cosmetics [231] and oral forms. The use of alginate hydrogels for tissue localization, however, is now attracting much interest. Alginate and chitosan, the ionic complexation agent, are both frequently employed in drug delivery applications. Chitin, from which chitosan originates, serves as one of the most common natural polymers in nature. Its pH is estimated to be 6.5, and it has characteristics defined by the existence of (1,4) linked -D-glucosamine. N-acetyl-D-glucosamine is frequently discovered in commercial goods at a 20% concentration, with -D-glucosamine making up the remaining 80% [88]. Chitosan is a cationic polymer that has been extensively employed in a variety of fields, including food, pharmaceuticals, biomedicine, and cosmetics, due to its high biocompatibility and numerous favorable properties [229]. Patents for various hydrogel formulations are listed in Table 3.

## 8. Conclusions and Future Prospects

Finally, as the circumstances of injuries to soft tissues and healing become more readily evident and as the invention of materials and processing techniques continues sodium-alginate-based hydrogels have become applied in a variety of domains of soft tissue engineering. Owing in a significant way to the spread of 3D bioprinting technology, sodium alginate hydrogels are in high demand for utilization in soft tissue engineering. These hydrogels can print precise geometric patterns and heterogeneous 3D tissues that mimic the structure and function of their respective tissues. These qualities provide bioprinting technology with an edge over traditional methods of manufacturing functional tissues and organs and provide the scientific foundation for the direct printing of functional soft tissues and organs. Soft tissue engineering has come a long way thanks largely to the many studies carried out during recent years on the topic of the 3D bioprinting of soft tissue, several of which have yielded positive findings. However, there are significant barriers to utilizing active organs with an integrated structure/function in therapeutic applications. The spatial distribution of the components that make up naturally occurring soft tissues, such as cell populations and intercellular matrices, and the interaction processes between them, can be exceedingly complicated. Alginate is a multifunctional medium with applications in tissue engineering and cell culture. There are several alternative formulations still in development for creating 3D alginate and hydrogel for use; however, hydrogel will be the preferred way to study tissue cell proliferation and differentiation mechanisms. This is not an explanation to avoid using 3D cell or tissue systems in domains such as tissue engineering and drug discovery but, rather, an opening to improve and individualize cellular structure testing. Hydrogel technology based on sodium alginate is in its infancy when it comes to the bionic production of tissue-built working organs, and many obstacles remain to be overcome. However, many discoveries are appearing as technology develops, and the existing problems are being addressed one by one. The3D bioprinting of functional tissues and organs in vivo with sodium-alginate-based hydrogel is expected to advance to the point of clinical application within the next few years. The goal of developing cutting-edge 3D bionic manufacturing techniques for functioning tissues as well as organs is to help with the repair and regeneration of damaged organs and to fix a wide range of tissue abnormalities.

## Figures and Tables

**Table 1:** Drug delivery techniques based on natural polymers.

Hydrogel Source	Additional Components	Synthesis Method	Loaded Drug	Ref
Chitosan	—	Formaldehyde crosslinking	DOX/5-FU	[54]
SA	Carbon nanotube whiskers	Ionic crosslinking	Metronidazole	[55]
SA	Polyvinyl alcohol/benzeneboronic acid	Ionic crosslinking	Proteins	[56]
SA	—	Ionic crosslinking	Iohexol	[57]
SA	Polyvinyl pyrrolidone	Ionic crosslinking	Exosomes	[58]
Hyaluronic acid	Gelatin	UV radiation	Epigallocatechin3-gallate	[59]

**Table 2:** The table below is a summary of the main distinctions between solid- and solution-state NMR [140].

	Solid-State NMR	Solution-State NMR
Type of sample	All physical states are possible	Only hydrolyzed gels
Sample preparation	Preparation (levels of hydration) is straightforward and manageable	Acid hydrolysis makes the preparation process lengthy
Restoration of samples	Yes	No
Limitations concerning hydrogels	Low sensitivity and resolution	Resolution depends on the solubility
Obtained information	Structure and dynamics of intact hydrogel	Chemical structure and composition

**Table 3:** List of patents for various hydrogel formulations [232].

S. No	Patent No./Country	Title	Disease/Problem	Details
1	US10799696B2 United States	Stimulating the nasolacrimal gland with a polymer formulation	Dry eye	The hydrogel formulation (made via a UV crosslinking technique) allows therapeutic electrical stimulation of the lacrimal gland, nasal, or sinus tissue to produce tears and treat dry eyes.
2	US20200085733A1 United States	Formulations of hypotonic hydrogels for improved delivery of therapeutics to mucosal surfaces	Used for diagnosis, prevention, and treatment by inserting into the vagina or colorectum	A polymeric hydrogel (poloxamers) in water acts as a plug and/or is delivered to a mucosal/epithelial surface for therapeutic, preventative, or diagnostic reasons.
3	CN105209016B China	Matrix hydrogel polymers for cell transport that are biocompatible	Provides cells with a stable environment that promotes their survival and activity	Hydrogel polymer matrices are biocompatible, bioabsorbable, and release cells at the place of application, allowing for localized and precise distribution.
4	US20180023049A1 United States	Hydrogel formulations without injection for controlled release	Experimenting with cultured cells	Solutions of synthetic peptide hydrogels with a pH of about 3.5and an osmolality in the isotonic range.
5	US20200360281A1 United States	An interstitial thermo-responsive hydrogel for the treatment of solid tumor malignancies	Solid tumor intra-tumoral chemotherapy	The injectable thermo-responsive hydrogel formed by crosslinking chitosan and genipin into an interpenetrating scaffold can efficiently integrate chemotherapeutic medicines without compromising the hydrogel’s inherent thermo-responsiveness.
6	JP6293254B2 Japan	Crosslinked hydrophilic coating on a silicone hydrogel lens	Corneal lenses	Contact lenses with a silicone hydrogel coating and a non-silicone hydrogel that is a crosslinked polymer of one or more crosslinkable components and a crosslinked carboxyl-containing polymer material are known as coated silicone hydrogel contact lenses.

## Data Availability

Data will be available on request.

## References

[1] Griffith L.G., Naughton G. (2002). Tissue engineering—Current challenges and expanding opportunities. Science.

[2] Zhang L., Fu L., Zhang X., Chen L., Cai Q., Yang X. (2021). Hierarchical and heterogeneous hydrogel system as a promising strategy for diversified interfacial tissue regeneration. Biomater. Sci..

[3] Atala A., Kasper F.K., Mikos A.G. (2012). Engineering complex tissues. Sci. Transl. Med..

[4] Zhang Y.S., Yue K., Aleman J., Mollazadeh-Moghaddam K., Bakht S.M., Yang J., Jia W., Dell’Erba V., Assawes P., Shin S.R. (2017). 3D bioprinting for tissue and organ fabrication. Ann. Biomed. Eng..

[5] Jian H., Wang M., Wang S., Wang A., Bai S. (2018). 3D bioprinting for cell culture and tissue fabrication. Bio-Des. Manuf..

[6] Slaughter B.V., Khurshid S.S., Fisher O.Z., Khademhosseini A., Peppas N.A. (2009). Hydrogels in regenerative medicine. Adv. Mater..

[7] Hu W., Wang Z., Xiao Y., Zhang S., Wang J. (2019). Advances in crosslinking strategies of biomedical hydrogels. Biomater. Sci..

[8] Pahlevanzadeh F., Mokhtari H., Bakhsheshi-Rad H.R., Emadi R., Kharaziha M., Valiani A., Poursamar S.A., Ismail A.F., RamaKrishna S., Berto F. (2020). Recent trends in three-dimensional bioinks based on alginate for biomedical applications. Materials.

[9] Tarassoli S.P., Jessop Z.M., Jovic T., Hawkins K., Whitaker I.S. (2021). Candidate bioinks for extrusion 3D bioprinting—A systematic review of the literature. Front. Bioeng. Biotechnol..

[10] Murab S., Gupta A., Włodarczyk-Biegun M.K., Kumar A., van Rijn P., Whitlock P., Han S.S., Agrawal G. (2022). Alginate based hydrogel inks for 3D bioprinting of engineered orthopedic tissues. Carbohyd. Polym..

[11] Gurikov P., Smirnova I. (2018). Non-conventional methods for gelation of alginate. Gels.

[12] Schütz K., Placht A.M., Paul B., Brüggemeier S., Gelinsky M., Lode A. (2017). Three-dimensional plotting of a cell-laden alginate/methylcellulose blend: Towards biofabrication of tissue engineering constructs with clinically relevant dimensions. J. Tissue Eng. Regenerat. Med..

[13] Li H., Tan C., Li L. (2018). Review of 3D printable hydrogels and constructs. Mater. Des..

[14] Hurtado A., Aljabali A.A., Mishra V., Tambuwala M.M., Serrano-Aroca Á. (2022). Alginate: Enhancement strategies for advanced applications. Int. J. Mol. Sci..

[15] Jin Y., Compaan A., Bhattacharjee T., Huang Y. (2016). Granular gel support-enabled extrusion of three-dimensional alginate and cellular structures. Biofabrication.

[16] Fernando I.S., Kim D., Nah J.W., Jeon Y.J. (2019). Advances in functionalizing fucoidans and alginates (bio) polymers by structural modifications: A review. Chem. Eng. J..

[17] Javed R., Shah L.A., Sayed M., Khan M.S. (2018). Uptake of heavy metal ions from aqueous media by hydrogels and their conversion to nanoparticles for generation of a catalyst system: Two-fold application study. RSC Adv..

[18] Richardson T., Barner S., Candiello J., Kumta P.N., Banerjee I. (2016). Capsule stiffness regulates the efficiency of pancreatic differentiation of human embryonic stem cells. Acta Biomater..

[19] Shea L.D., Woodruff T.K., Shikanov A. (2014). Bioengineering the ovarian follicle microenvironment. Annu. Rev. Biomed. Eng.

[20] Tomaszewski C.E., DiLillo K.M., Baker B.M., Arnold K.B., Shikanov A. (2021). Sequestered cell-secreted extracellular matrix proteins improve murine folliculogenesis and oocyte maturation for fertility preservation. Acta Biomater..

[21] Laronda M.M., Duncan F.E., Hornick J.E., Xu M., Pahnke J.E., Whelan K.A., Shea L.D., Woodruff T.K. (2014). Alginate encapsulation supports the growth and differentiation of human primordial follicles within ovarian cortical tissue. J. Assist. Reprod. Genet..

[22] Avila-Rodríguez D., Paisano-Cerón K., Valdovinos-Ramírez I., Solano-Agama C., Ortiz-Plata A., Mendoza-Garrido M.E. (2016). Three-dimensional alginate-bead culture of human pituitary adenoma cells. J. Vis. Experim..

[23] Rider P., Kačarević Ž.P., Alkildani S., Retnasingh S., Barbeck M. (2018). Bioprinting of tissue engineering scaffolds. J. Tissue Eng..

[24] Szekalska M., Puciłowska A., Szymańska E., Ciosek P., Winnicka K. (2016). Alginate: Current use and future perspectives in pharmaceutical and biomedical applications. Int. J. Polym. Sci..

[25] Chen C., Xi Y., Weng Y. (2022). Recent advances in cellulose-based hydrogels for tissue engineering applications. Polymers.

[26] Nascimento D.M., Nunes Y.L., Figueirêdo M.C., de Azeredo H.M., Aouada F.A., Feitosa J.P., Rosa M.F., Dufresne A. (2018). Nanocellulose nanocomposite hydrogels: Technological and environmental issues. Green Chem..

[27] Aram E., Mehdipour-Ataei S. (2021). Carbon-based nanostructured composites for tissue engineering and drug delivery. Int. J. Polym. Mater. Polym. Biomater..

[28] Fernando I.P.S., Lee W., Han E.J., Ahn G. (2020). Alginate-based nanomaterials: Fabrication techniques, properties, and applications. Chem. Eng. J..

[29] Abdelbasset W.K., Jasim S.A., Sharma S.K., Margiana R., Bokov D.O., Obaid M.A., Hussein B.A., Lafta H.A., Jasim S.F., Mustafa Y.F. (2022). Alginate-based hydrogels and tubes, as biological macromolecule-based platforms for peripheral nerve tissue engineering: A review. Ann. Biomed. Eng..

[30] Srivastava V.K., Jain P.K., Kumar P., Pegoretti A., Bowen C.R. (2020). Smart manufacturing process of carbon-based low-dimensional structures and fiber-reinforced polymer composites for engineering applications. J. Mater. Eng. Perform..

[31] Ramiah P., Du Toit L.C., Choonara Y.E., Kondiah P.P., Pillay V. (2020). Hydrogel-based bioinks for 3D bioprinting in tissue regeneration. Front. Mater..

[32] Mali P., Sherje A.P. (2022). Cellulose nanocrystals: Fundamentals and biomedical applications. Carbohyd. Polym..

[33] Das S., Basu B. (2019). An overview of hydrogel-based bioinks for 3D bioprinting of soft tissues. J. Indian Inst. Sci..

[34] Szychlinska M.A., Bucchieri F., Fucarino A., Ronca A., D’Amora U. (2022). Three-Dimensional Bioprinting for Cartilage Tissue Engineering: Insights into Naturally-derived Bioinks from land and marine sources. J. Funct. Biomater..

[35] Zdiri K., Cayla A., Elamri A., Erard A., Salaun F. (2022). Alginate-Based Bio-Composites and Their Potential Applications. J. Funct. Biomater..

[36] Selvan N.K., Shanmugarajan T.S., Uppuluri V.N.V.A. (2020). Hydrogel based scaffolding polymeric biomaterials: Approaches towards skin tissue regeneration. J. Drug Deliv. Sci. Technol..

[37] Ćorković I., Pichler A., Šimunović J., Kopjar M. (2021). Hydrogels: Characteristics and application as delivery systems of phenolic and aroma compounds. Foods.

[38] Rahmani Del Bakhshayesh A., Annabi N., Khalilov R., Akbarzadeh A., Samiei M., Alizadeh E., Alizadeh-Ghodsi M., Davaran S., Montaseri A. (2018). Recent advances on biomedical applications of scaffolds in wound healing and dermal tissue engineering. Artif. Cells Nanomed. Biotechnol..

[39] Peppas N.A. (2010). Biomedical Applications of Hydrogels Handbook.

[40] Lodhi R.S., Das S., Das P. (2023). Recent Advances in Polymer Hydrogels for Agricultural Applications. Novel Polymeric Materials for Environmental Applications.

[41] Russo E., Villa C. (2019). Poloxamer hydrogels for biomedical applications. Pharmaceutics.

[42] Yu Y., Cheng Y., Tong J., Zhang L., Wei Y., Tian M. (2021). Recent advances in thermo-sensitive hydrogels for drug delivery. J. Mater. Chem..

[43] Sun X., Agate S., Salem K.S., Lucia L., Pal L. (2020). Hydrogel-based sensor networks: Compositions, properties, and applications—A review. ACS Appl. Bio Mater..

[44] Gerlach G., Arndt K. (2013). Hydrogel Sensors and Actuators Volume.

[45] Byju A.G., Kulkarni A., Gundiah N. Mechanics of gelatin and elastin-based hydrogels as tissue engineered constructs. Proceedings of the 13th International Conference Fracture.

[46] Oyen M.L. (2013). Mechanical characterisation of hydrogel materials. Int. Mater. Rev..

[47] Hua J., Ng P.F., Fei B. (2018). High-strength hydrogels: Microstructure design, characterization, and applications. J. Polym. Sci..

[48] Maitra J., Shukla V.K. (2014). Cross-linking in hydrogels-a review. Am. J. Polym. Sci..

[49] Weber L.M., Lopez C.G., Anseth K.S. (2009). Effects of PEG hydrogel crosslinking density on protein diffusion and encapsulated islet survival and function. J. Biomed. Mater. Res..

[50] Sung H.W., Huang D.M., Chang W.H., Huang R.N., Hsu J.C. (1999). Evaluation of gelatin hydrogel crosslinked with various crosslinking agents as bioadhesives: In vitro study. J. Biomed. Mater. Res..

[51] Parhi R. (2017). Cross-linked hydrogel for pharmaceutical applications: A review. Adv. Pharm. Bull..

[52] Gul K., Gan R.Y., Sun C.X., Jiao G., Wu D.T., Li H.B., Kenaan A., Corke H., Fang Y.P. (2022). Recent advances in the structure, synthesis, and applications of natural polymeric hydrogels. Crit. Rev. Food Sci. Nutr..

[53] Coviello T., Matricardi P., Marianecci C., Alhaique F. (2007). Polysaccharide hydrogels for modified release formulations. J. Control. Release.

[54] Omrani M., Naimi-Jamal M.R., Far B.F. (2022). The design of multi-responsive nanohydrogel networks of chitosan for controlled drug delivery. Carbohyd. Polym..

[55] Dubashynskaya N.V., Petrova V.A., Romanov D.P., Skorik Y.A. (2022). pH-Sensitive Drug Delivery System Based on Chitin Nanowhiskers–Sodium Alginate Polyelectrolyte Complex. Materials.

[56] MassanaRoquero D., Bollella P., Katz E., Melman A. (2021). Controlling porosity of calcium alginate hydrogels by interpenetrating polyvinyl alcohol–diboronate polymer network. ACS Appl. Polym. Mater..

[57] Ren M., Li N., Jiang X., Liu X., Zou A. (2022). Efficient oral delivery of water-soluble CT contrast agent using an W1/O/W2 alginate hydrogel matrix. Colloids Surf..

[58] Sadeghian-Nodoushan F., Nikukar H., Soleimani M., Jalali-Jahromi A., Hosseinzadeh S., Khojasteh A. (2022). A smart magnetic hydrogel containing exosome promotes osteogenic commitment of human adipose-derived mesenchymal stem cells. Iran. J. Basic Med. Sci..

[59] He Z., Luo H., Wang Z., Chen D., Feng Q., Cao X. (2023). Injectable and tissue adhesive EGCG-laden hyaluronic acid hydrogel depot for treating oxidative stress and inflammation. Carbohyd. Polym..

[60] Ahmadi F., Oveisi Z., Samani S.M., Amoozgar Z. (2015). Chitosan based hydrogels: Characteristics and pharmaceutical applications. Res. Pharm. Sci..

[61] Casadidio C., Peregrina D.V., Gigliobianco M.R., Deng S., Censi R., Di Martino P. (2019). Chitin and chitosans: Characteristics, eco-friendly processes, and applications in cosmetic science. Mar. Drugs.

[62] Rinaudo M. (2006). Chitin and chitosan: Properties and applications. Progress Polym. Sci..

[63] Singh S., Chunglok W., Nwabor O.F., Chulrik W., Jansakun C., Bhoopong P. (2023). Porous biodegradable sodium alginate composite fortified with *Hibiscus Sabdariffa* L. Calyx extract for the multifarious biological applications and extension of climacteric fruit shelf-life. J. Polym. Environ..

[64] Tian B., Hua S., Tian Y., Liu J. (2020). Chemical and physical chitosan hydrogels as prospective carriers for drug delivery: A review. J. Mater. Chem..

[65] Fu L.H., Qi C., Ma M.G., Wan P. (2019). Multifunctional cellulose-based hydrogels for biomedical applications. J. Mater. Chem..

[66] Rusu D., Ciolacu D., Simionescu B.C. (2019). Cellulose-based hydrogels in tissue engineering applications. Cell. Chem. Technol..

[67] Habibi Y., Lucia L.A., Rojas O.J. (2010). Cellulose nanocrystals: Chemistry, self-assembly, and applications. Chem. Rev..

[68] Ciolacu D.E., Nicu R., Ciolacu F. (2020). Cellulose-based hydrogels as sustained drug-delivery systems. Materials.

[69] Pettignano A., Charlot A., Fleury E. (2019). Carboxyl-functionalized derivatives of carboxymethyl cellulose: Towards advanced biomedical applications. Polym. Rev..

[70] Reif B., Ashbrook S.E., Emsley L., Hong M. (2021). Solid-state NMR spectroscopy. Nat. Rev. Methods Prim..

[71] Borisov A.S., Hazendonk P., Hayes P.G. (2010). Solid-state nuclear magnetic resonance spectroscopy: A review of modern techniques and applications for inorganic polymers. J. Inorg. Organomet. Polym. Mater..

[72] Brown S.P. (2018). Advanced solid-state NMR methods for characterising structure and self-assembly in supramolecular chemistry, polymers and hydrogels. Curr. Opin. Colloid Int. Sci..

[73] Mathur A.M., Scranton A.B. (1996). Characterization of hydrogels using nuclear magnetic resonance spectroscopy. Biomaterials.

[74] Weingarth M., Baldus M. (2013). Solid-state NMR-based approaches for supramolecular structure elucidation. Accounts Chem. Res..

[75] El Hariri El Nokab M., Sebakhy K.O. (2021). Solid State NMR Spectroscopy a Valuable Technique for Structural Insights of Advanced Thin Film Materials: A Review. Nanomaterials.

[76] Polenova T., Gupta R., Goldbourt A. (2015). Magic angle spinning NMR spectroscopy: A versatile technique for structural and dynamic analysis of solid-phase systems. Anal. Chem..

[77] Ganji F., Abdekhodaie M.J., Ramazani SA A. (2007). Gelation time and degradation rate of chitosan-based injectable hydrogel. J. Sol-Gel Sci. Technol..

[78] Cho J., Heuzey M.C., Bégin A., Carreau P.J. (2005). Physical gelation of chitosan in the presence of β-glycerophosphate: The effect of temperature. Biomacromolecules.

[79] Xu W., Shen R., Yan Y., Gao J. (2017). Preparation and characterization of electrospun alginate/PLA nanofibers as tissue engineering material by emulsion eletrospinning. J. Mech. Biomed. Mater..

[80] Nishiyama Y., Nakamura M., Henmi C., Yamaguchi K., Mochizuki S., Nakagawa H., Takiura K. (2009). Development of a three-dimensional bioprinter: Construction of cell supporting structures using hydrogel and state-of-the-art inkjet technology. J. Biomech. Eng..

[81] Luo Y., Luo G., Gelinsky M., Huang P., Ruan C. (2009). 3D bioprinting scaffold using alginate/polyvinyl alcohol bioinks. Mater. Let..

[82] Caykara T., Demirci S., Eroğlu M.S., Güven O. (2005). Poly (ethylene oxide) and its blends with sodium alginate. Polymer.

[83] Yamaoka H., Asato H., Ogasawara T., Nishizawa S., Takahashi T., Nakatsuka T., Koshima I., Nakamura K., Kawaguchi H., Chung U.I. (2006). Cartilage tissue engineering using human auricular chondrocytes embedded in different hydrogel materials. J. Biomed. Mater..

[84] Kuijpers A.J., Engbers G.H., Krijgsveld J., Zaat S.A., Dankert J., Feijen J. (2000). Cross-linking and characterisation of gelatin matrices for biomedical applications. J. Biomat. Sci..

[85] Daamen W.F., Hafmans T., Veerkamp J.H., Van Kuppevelt T.H. (2001). Comparison of five procedures for the purification of insoluble elastin. Biomaterials.

[86] Zawko S.A., Suri S., Truong Q., Schmidt C.E. (2009). Photopatterned anisotropic swelling of dual-crosslinked hyaluronic acid hydrogels. Acta Biomater..

[87] Abbah S.A., Lu W.W., Chan D., Cheung K.M.C., Liu W.G., Zhao F., Li Z.Y., Leong J.C.Y., Luk K.D.K. (2008). Osteogenic behavior of alginate encapsulated bone marrow stromal cells: An in vitro study. J. Mater. Sci..

[88] Gerlach G., Arndt K.F. (2009). Hydrogel Sensors and Actuators: Engineering and Technology.

[89] Sathaye S., Mbi A., Sonmez C., Chen Y., Blair D.L., Schneider J.P., Pochan D.J. (2015). Rheology of peptide-and protein-based physical hydrogels: Are everyday measurements just scratching the surface. Nanomed. Nanobiotechnol..

[90] Sahan A.Z., Baday M., Patel C.B. (2022). Biomimetic Hydrogels in the Study of Cancer Mechanobiology: Overview, Biomedical Applications, and Future Perspectives. Gels.

[91] Kothale D., Verma U., Dewangan N., Jana P., Jain A., Jain D. (2020). Alginate as promising natural polymer for pharmaceutical, food, and biomedical applications. Curr. Drug Deliv..

[92] Yang J.S., Xie Y.J., He W. (2011). Research progress on chemical modification of alginate: A review. Carbohyd. Polym..

[93] Reakasame S., Boccaccini A.R. (2018). Oxidized alginate-based hydrogels for tissue engineering applications: A review. Biomacromolecules.

[94] Gao C., Liu M., Chen J., Zhang X. (2009). Preparation and controlled degradation of oxidized sodium alginate hydrogel. Polym. Degrad. Stab..

[95] Emami Z., Ehsani M., Zandi M., Foudazi R. (2018). Controlling alginate oxidation conditions for making alginate-gelatin hydrogels. Carbohyd. Polym..

[96] Baniasadi H., Mashayekhan S., Fadaoddini S., Haghirsharifzamini Y. (2016). Design, fabrication and characterization of oxidized alginate–gelatin hydrogels for muscle tissue engineering applications. J. Biomater. Appl..

[97] Barceló X., Eichholz K.F., Garcia O., Kelly D.J. (2022). Tuning the degradation rate of alginate-based bioinks for bioprinting functional cartilage tissue. Biomedicines.

[98] Heo D.N., Alioglu M.A., Wu Y., Ozbolat V., Ayan B., Dey M., Kang Y., Ozbolat I.T. (2020). 3D bioprinting of carbohydrazide-modified gelatin into microparticle-suspended oxidized alginate for the fabrication of complex-shaped tissue constructs. ACS Appl. Mater. Int..

[99] Matsumura K., Rajan R. (2021). Oxidized polysaccharides as green and sustainable biomaterials. Curr. Org. Chem..

[100] Naghizadeh Z., Karkhaneh A., Khojasteh A. (2018). Self-crosslinking effect of chitosan and gelatin on alginate-based hydrogels: Injectable in situ forming scaffolds. Mater. Sci. Eng..

[101] Distler T., Polley C., Shi F., Schneidereit D., Ashton M.D., Friedrich O., Kolb J.F., Hardy J.G., Detsch R., Seitz H. (2021). Electrically conductive and 3D-printable oxidized alginate-gelatin polypyrrole: PSS hydrogels for tissue engineering. Adv. Healthc. Mater..

[102] Öztürk E., Stauber T., Levinson C., Cavalli E., Arlov Ø., Zenobi-Wong M. (2020). Tyrosinase-crosslinked, tissue adhesive and biomimetic alginate sulfate hydrogels for cartilage repair. Biomed. Mater..

[103] Malaeb W., Bahmad H.F., Abou-Kheir W., Mhanna R. (2019). The sulfation of biomimetic glycosaminoglycan substrates controls binding of growth factors and subsequent neural and glial cell growth. Biomater. Sci..

[104] Kfoury G., El Habbaki V., Malaeb W., Weaver S., Momotenko D., Mhanna R. (2020). Alginate sulfate substrates control growth factor binding and growth of primary neurons: Toward engineered 3D neural networks. Adv. Biosyst..

[105] Freeman I., Cohen S. (2009). The influence of the sequential delivery of angiogenic factors from affinity-binding alginate scaffolds on vascularization. Biomaterials.

[106] Gong J.P., Katsuyama Y., Kurokawa T., Osada Y. (2003). Double-network hydrogels with extremely high mechanical strength. Adv. Mater..

[107] Chen Y., Dong K., Liu Z., Xu F. (2012). Double network hydrogel with high mechanical strength: Performance, progress and future perspective. Sci. China Technol. Sci..

[108] Zhang M., Ren X., Duan L., Gao G. (2018). Joint double-network hydrogels with excellent mechanical performance. Polymer.

[109] Chen C., Zhu Y., Bao H., Zhao P., Jiang H., Peng L., Yang X., Li C. (2011). Solvent-assisted poly (vinyl alcohol) gelated crystalline colloidal array photonic crystals. Soft Matter.

[110] Wang X., Qiu Y., Chen G., Chu Z., Shadike A., Chen C., Chen C., Zhu Z. (2020). Self-healable poly (vinyl alcohol) photonic crystal hydrogel. ACS Appl. Polym. Mater..

[111] Elango J., Zamora-Ledezma C., Negrete-Bolagay D., Aza P.N.D., Gómez-López V.M., López-González I., Belén Hernández A., De Val J.E.M.S., Wu W. (2022). Retinol-Loaded Poly (vinyl alcohol)-Based Hydrogels as Suitable Biomaterials with Antimicrobial Properties for the Proliferation of Mesenchymal Stem Cells. Int. J. Mol. Sci..

[112] Chen W., Li N., Ma Y., Minus M.L., Benson K., Lu X., Wang X., Ling X., Zhu H. (2019). Superstrong and tough hydrogel through physical cross-linking and molecular alignment. Biomacromolecules.

[113] Xu C., Zhang X., Liu S., Zhao X., Geng C., Wang L., Xia Y. (2021). Selected phase separation renders high strength and toughness to polyacrylamide/alginate hydrogels with large-scale cross-linking zones. ACS Appl. Mater. Interfaces.

[114] Zhao D., Feng M., Zhang L., He B., Chen X., Sun J. (2021). Facile synthesis of self-healing and layered sodium alginate/polyacrylamide hydrogel promoted by dynamic hydrogen bond. Carbohyd. Polym..

[115] Zhang Z., Lin T., Li S., Chen X., Que X., Sheng L., Hu Y., Peng J., Ma H., Li J. (2022). Polyacrylamide/Copper-Alginate Double Network Hydrogel Electrolyte with Excellent Mechanical Properties and Strain-Sensitivity. Macromol. Biosci..

[116] Xu R., Ma S., Lin P., Yu B., Zhou F., Liu W. (2017). High strength astringent hydrogels using protein as the building block for physically cross-linked multi-network. ACS Appl. Mater. Int..

[117] Taki M., Yamashita T., Yatabe K., Vogel V. (2019). Mechano-chromic protein–polymer hybrid hydrogel to visualize mechanical strain. Soft Matter.

[118] Aarstad O., Strand B.L., Klepp-Andersen L.M., Skjåk-Bræk G. (2013). Analysis of G-block distributions and their impact on gel properties of in vitro epimerized mannuronan. Biomacromolecules.

[119] Murguía-Flores D.A., Bonilla-Ríos J., Canales-Fiscal M.R., Sánchez-Fernández A. (2016). Protein adsorption through Chitosan–Alginate membranes for potential applications. Chem. Central J..

[120] Jiang Y.Y., Zhu Y.J., Li H., Zhang Y.G., Shen Y.Q., Sun T.W., Chen F. (2017). Preparation and enhanced mechanical properties of hybrid hydrogels comprising ultralong hydroxyapatite nanowires and sodium alginate. J. Colloid Int. Sci..

[121] Toti U.S., Aminabhavi T.M. (2004). Different viscosity grade sodium alginate and modified sodium alginate membranes in pervaporation separation of water+ acetic acid and water+ isopropanol mixtures. J. Membr. Sci..

[122] Davis T.A., Llanes F., Volesky B., Diaz-Pulido G., McCook L., Mucci A. (2003). 1 H-NMR study of Na alginates extracted from Sargassum spp. in relation to metal biosorption. Appl. Biochem. Biotechnol..

[123] Naranjo-Alcazar R., Bendix S., Groth T., Gallego Ferrer G. (2023). Research Progress in Enzymatically Cross-Linked Hydrogels as Injectable Systems for Bioprinting and Tissue Engineering. Gels.

[124] Lee K.Y., Mooney D.J. (2012). Alginate: Properties and biomedical applications. Prog. Polym. Sci..

[125] Sun J., Tan H. (2013). Alginate-based biomaterials for regenerative medicine applications. Materials.

[126] Jhon M.S., Andrade J.D. (1973). Water and hydrogels. J. Biomed. Mater. Res..

[127] Böckmann A., Gardiennet C., Verel R., Hunkeler A., Loquet A., Pintacuda G., Emsley L., Meier B.H., Lesage A. (2009). Characterization of different water pools in solid-state NMR protein samples. J. Biomol. NMR.

[128] Mandal A., van der Wel P.C. (2016). MAS 1H NMR probes freezing point depression of water and liquid-gel phase transitions in liposomes. Biophys. J..

[129] Gun’ko V.M., Savina I.N., Mikhalovsky S.V. (2017). Properties of water bound in hydrogels. Gels.

[130] Gun’ko V.M., Turov V.V., Bogatyrev V.M., Zarko V.I., Leboda R., Goncharuk E.V., Novza A.A., Turov A.V., Chuiko A.A. (2005). Unusual properties of water at hydrophilic/hydrophobic interfaces. Adv. Colloid Int. Sci..

[131] Gun’Ko V.M., Zarko V.I., Goncharuk E.V., Andriyko L.S., Turov V.V., Nychiporuk Y.M., Leboda R., Skubiszewska-Zięba J., Gabchak A.L., Osovskii V.D. (2007). TSDC spectroscopy of relaxational and interfacial phenomena. Adv. Colloid Int. Sci..

[132] El Hariri El Nokab M., Habib M.H., Alassmy Y.A., Abduljawad M.M., Alshamrani K.M., Sebakhy K.O. (2022). Solid state NMR a powerful technique for investigating sustainable/renewable cellulose-based materials. Polymers.

[133] Wang T., Hong M. (2016). Solid-state NMR investigations of cellulose structure and interactions with matrix polysaccharides in plant primary cell walls. J. Exp. Bot..

[134] Munekata P.E., Gullón B., Pateiro M., Tomasevic I., Domínguez R., Lorenzo J.M. (2020). Natural antioxidants from seeds and their application in meat products. Antioxidants.

[135] Fu R., Wang X., Li C., Santiago-Miranda A.N., Pielak G.J., Tian F. (2011). In situ structural characterization of a recombinant protein in native *Escherichia coli* membranes with solid-state magic-angle-spinning NMR. J. Am. Chem. Soc..

[136] Li B., Xu L., Wu Q., Chen T., Sun P., Jin Q., Ding D., Wang X., Xue G., Shi A.C. (2007). Various types of hydrogen bonds, their temperature dependence and water polymer interaction in hydrated poly (acrylic acid) as revealed by 1H solid-state NMR spectroscopy. Macromolecules.

[137] van der Wel P.C. (2018). New applications of solid-state NMR in structural biology. Emerg. Topics Life Sci..

[138] Weingarth M., Ader C., Melquiond A.S., Nand D., Pongs O., Becker S., Bonvin A.M., Baldus M. (2012). Supramolecular structure of membrane-associated polypeptides by combining solid-state NMR and molecular dynamics simulations. Biophys. J..

[139] Wallace M., Iggo J.A., Adams D.J. (2015). Using solution state NMR spectroscopy to probe NMR invisible gelators. Soft Matter.

[140] Zia K., Siddiqui T., Ali S., Farooq I., Zafar M.S., Khurshid Z. (2019). Nuclear magnetic resonance spectroscopy for medical and dental applications: A comprehensive review. Eur. J. Dent..

[141] Jiang J., Cui H., Cao Y. (2014). Preparation and property studies of Zn 3 P 2/calcium alginate. Nano.

[142] Leal D., Matsuhiro B., Rossi M., Caruso F. (2008). FT-IR spectra of alginic acid block fractions in three species of brown seaweeds. Carbohyd. Res..

[143] Pereira L., Critchley A.T., Amado A.M., Ribeiro-Claro P.J. (2009). A comparative analysis of phycocolloids produced by underutilized versus industrially utilized carrageenophytes (Gigartinales, Rhodophyta). J. Appl. Phycol..

[144] Salomonsen T., Jensen H.M., Stenbæk D., Engelsen S.B. (2008). Chemometric prediction of alginate monomer composition: A comparative spectroscopic study using IR, Raman, NIR and NMR. Carbohyd. Polym..

[145] Ivleva N.P., Wagner M., Horn H., Niessner R., Haisch C. (2009). Towards a nondestructive chemical characterization of biofilm matrix by Raman microscopy. Anal. Bioanalyt. Chem..

[146] Aroca R. (2006). Surface-Enhanced Vibrational Spectroscopy.

[147] Fleischmann M., Hendra P.J., McQuillan A.J. (1974). Raman spectra of pyridine adsorbed at a silver electrode. Chem. Phys. Lett..

[148] Xie H., Becraft E.J., Baughman R.H., Dalton A.B., Dieckmann G.R. (2008). Ranking the affinity of aromatic residues for carbon nanotubes by using designed surfactant peptides. J. Pept. Sci..

[149] Schmid T., Messmer A., Yeo B.S., Zhang W., Zenobi R. (2008). Towards chemical analysis of nanostructures in biofilms II: Tip-enhanced Raman spectroscopy of alginates. Anal. Bioanalyt. Chem..

[150] Atkins E.D.T., Mackie W., Parker K.D., Smolko E.E. (1971). Crystalline structures of poly-D-mannuronic and poly-L-guluronic acids. J. Polym. Sci..

[151] Atkins E.D.T., Mackie W., Smolko E.E. (1970). Crystalline structures of alginic acids. Nature.

[152] Landa N., Miller L., Feinberg M.S., Holbova R., Shachar M., Freeman I., Cohen S., Leor J. (2008). Effect of injectable alginate implant on cardiac remodeling and function after recent and old infarcts in rat. Circulation.

[153] Gilbert S.F. (2001). Ecological developmental biology: Developmental biology meets the real world. Dev. Biol..

[154] Krentz K.J., Nebel R.L., Canseco R.S., McGilliard M.L. (1993). In vitro and in vivo development of mouse morulae encapsulated in 2% sodium alginate or 0.1% poly-l-lysine. Theriogenology.

[155] Groeber F., Holeiter M., Hampel M., Hinderer S., Schenke-Layland K. (2011). Skin tissue engineering—In vivo and in vitro applications. Adv. Drug Deliv. Rev..

[156] Shin C.S., Kwak B., Han B., Park K. (2013). Development of an in vitro 3D tumor model to study therapeutic efficiency of an anticancer drug. Mol. Pharm..

[157] Patel H.R., Patel R.R., Patel L.D., Patel Y., Raval A. (2016). Preparation and in vitro characterization of non-effervescent floating delivery system of cefpodoxime proxetil. Pharmacophore.

[158] Al-Hatamleh M.A., Alshaer W., Hatmal M.M.M., Lambuk L., Ahmed N., Mustafa M.Z., Low S.C., Jaafar J., Ferji K., Six J.L. (2022). Applications of alginate-based nanomaterials in enhancing the therapeutic effects of bee products. Front. Mol. Biosci..

[159] Liu W., Madry H., Cucchiarini M. (2022). Application of alginate hydrogels for next-generation articular cartilage regeneration. Int. J. Mol. Sci..

[160] Alarcin E., Bal-Öztürk A., Avci H., Ghorbanpoor H., Dogan Guzel F., Akpek A., Yesiltas G., Canak-Ipek T., Avci-Adali M. (2021). Current strategies for the regeneration of skeletal muscle tissue. Int. J. Mol. Sci..

[161] Rosiak P., Latanska I., Paul P., Sujka W., Kolesinska B. (2021). Modification of alginates to modulate their physic-chemical properties and obtain biomaterials with different functional properties. Molecules.

[162] Grijalvo S., Nieto-Díaz M., Maza R.M., Eritja R., Díaz D.D. (2019). Alginate hydrogels as scaffolds and delivery systems to repair the damaged spinal cord. Biotechnol. J..

[163] Rasool B.K.A., FAHMY S. (2013). Development of coated beads for oral controlled delivery of cefaclor: In vitro evaluation. Acta Pharm..

[164] Martinez A., Muniz E., Iglesias I., Teijon J.M., Blanco M.D. (2012). Enhanced preclinical efficacy of tamoxifen developed as alginate–cysteine/disulfide bond reduced albumin nanoparticles. Int. J. Pharm..

[165] Ab-Rahim S., Selvaratnam L., Raghavendran H.R.B., Kamarul T. (2013). Chondrocyte-alginate constructs with or without TGF-β1 produces superior extracellular matrix expression than monolayer cultures. Mol. Cell. Biochem..

[166] Hill E., Boontheekul T., Mooney D.J. (2006). Designing scaffolds to enhance transplanted myoblast survival and migration. Tissue Eng..

[167] Rowley J.A., Mooney D.J. (2002). Alginate type and RGD density control myoblast phenotype. J. Biomed. Mater. Res..

[168] Purcell E.K., Singh A., Kipke D.R. (2009). Alginate composition effects on a neural stem cell–seeded scaffold. Tissue Eng..

[169] Froelich A., Jakubowska E., Wojtyłko M., Jadach B., Gackowski M., Gadziński P., Napierała O., Ravliv Y., Osmałek T. (2023). Alginate-Based Materials Loaded with Nanoparticles in Wound Healing. Pharmaceutics.

[170] Siqueira P., Siqueira É., De Lima A.E., Siqueira G., Pinzón-Garcia A.D., Lopes A.P., Segura M.E.C., Isaac A., Pereira F.V., Botaro V.R. (2019). Three-dimensional stable alginate-nanocellulose gels for biomedical applications: Towards tunable mechanical properties and cell growing. Nanomaterials.

[171] Kawaguchi M., Fukushima T., Hayakawa T., Nakashima N., Inoue Y., Takeda S., Okamura K., Taniguchi K. (2006). Preparation of carbon nanotube-alginate nanocomposite gel for tissue engineering. Dent. Mater. J..

[172] De Silva R.T., Mantilaka M.M.M.G.P.G., Goh K.L., Ratnayake S.P., Amaratunga G.A.J., de Silva K.M. (2017). Magnesium oxide nanoparticles reinforced electrospun alginate-based nanofibrous scaffolds with improved physical properties. Int. J. Biomater..

[173] Golafshan N., Kharaziha M., Fathi M. (2017). Tough and conductive hybrid graphene-PVA: Alginate fibrous scaffolds for engineering neural construct. Carbon.

[174] Yildirim E.D., Yin X., Nair K., Sun W. (2008). Fabrication, characterization, and biocompatibility of single-walled carbon nanotube-reinforced alginate composite scaffolds manufactured using freeform fabrication technique. J. Biomed. Mater. Res..

[175] Wu Y., Lin Z.Y.W., Wenger A.C., Tam K.C., Tang X.S. (2018). 3D bioprinting of liver-mimetic construct with alginate/cellulose nanocrystal hybrid bioink. Bioprinting.

[176] Fan Y., Yue Z., Lucarelli E., Wallace G.G. (2020). Hybrid printing using cellulose nanocrystals reinforced GelMA/HAMA hydrogels for improved structural integration. Adv. Healthc. Mater..

[177] Temirel M., Hawxhurst C., Tasoglu S. (2021). Shape fidelity of 3D-bioprinted biodegradable patches. Micromachines.

[178] Markstedt K., Mantas A., Tournier I., Martínez Ávila H., Hagg D., Gatenholm P. (2015). 3D bioprinting human chondrocytes with nanocellulose–alginate bioink for cartilage tissue engineering applications. Biomacromolecules.

[179] Zhang X., Huang C., Zhao Y., Jin X. (2017). Preparation, and characterization of nanoparticle reinforced alginate fibers with high porosity for potential wound dressing application. RSC Adv..

[180] Seal B.L., Otero T.C., Panitch A.J.M.S. (2001). Polymeric biomaterials for tissue and organ regeneration. Mater. Sci. Eng..

[181] Catoira M.C., Fusaro L., Di Francesco D., Ramella M., Boccafoschi F. (2019). Overview of natural hydrogels for regenerative medicine applications. J. Mater. Sci..

[182] Wei Q., Zhou J., An Y., Li M., Zhang J., Yang S. (2023). Modification, 3D printing process and application of sodium alginate-based hydrogels in soft tissue engineering: A review. Int. J. Biol. Macromol..

[183] Wang G., Wang X., Huang L. (2017). Feasibility of chitosan-alginate (Chi-Alg) hydrogel used as scaffold for neural tissue engineering: A pilot study in vitro. Biotechnol. Biotechnol. Equip..

[184] Bushkalova R., Farno M., Tenailleau C., Duployer B., Cussac D., Parini A., Sallerin B., Fullana S.G. (2019). Alginate-chitosan PEC scaffolds: A useful tool for soft tissues cell therapy. Int. J. Pharm..

[185] Dahlmann J., Krause A., Möller L., Kensah G., Möwes M., Diekmann A., Martin U., Kirschning A., Gruh I., Dräger G. (2013). Fully defined in situ cross-linkable alginate and hyaluronic acid hydrogels for myocardial tissue engineering. Biomaterials.

[186] Pérez-Madrigal M.M., Shaw J.E., Arno M.C., Hoyland J.A., Richardson S.M., Dove A.P. (2020). Robust alginate/hyaluronic acid thiol–yne click-hydrogel scaffolds with superior mechanical performance and stability for load-bearing soft tissue engineering. Biomater. Sci..

[187] Wang Q.Q., Liu Y., Zhang C.J., Zhang C., Zhu P. (2019). Alginate/gelatin blended hydrogel fibers cross-linked by Ca^2+^ and oxidized starch: Preparation and properties. Mater. Sci. Eng..

[188] Di Giuseppe M., Law N., Webb B., Macrae R.A., Liew L.J., Sercombe T.B., Dilley R.J., Doyle B.J. (2018). Mechanical behavior of alginate-gelatin hydrogels for 3D bioprinting. J. Mech. Behav. Biomed. Mater..

[189] Yeo M., Lee J.S., Chun W., Kim G.H. (2016). An innovative collagen-based cell-printing method for obtaining human adipose stem cell-laden structures consisting of core–sheath structures for tissue engineering. Biomacromolecules.

[190] Lee H.J., Kim Y.B., Ahn S.H., Lee J.S., Jang C.H., Yoon H., Chun W., Kim G.H. (2015). A new approach for fabricating collagen/ECM-based bio-inks using pre-osteoblasts and human adipose stem cells. Adv. Healthc. Mater..

[191] Solovieva E.V., Fedotov A.Y., Mamonov V.E., Komlev V.S., Panteleyev A.A. (2018). Fibrinogen-modified sodium alginate as a scaffold material for skin tissue engineering. Biomed. Mater..

[192] Ahmed E.M., Aggor F.S., Awad A.M., El-Aref A.T. (2013). An innovative method for preparation of nanometal hydroxide superabsorbent hydrogel. Carbohyd. Polym..

[193] Candiello J., Singh S.S., Task K., Kumta P.N., Banerjee I. (2013). Early differentiation patterning of mouse embryonic stem cells in response to variations in alginate substrate stiffness. J. Biol. Eng..

[194] Xu M., Kreeger P.K., Shea L.D., Woodruff T.K. (2006). Tissue-engineered follicles produce live, fertile offspring. Tissue Eng..

[195] Kreeger P.K., Deck J.W., Woodruff T.K., Shea L.D. (2006). The in vitro regulation of ovarian follicle development using alginate-extracellular matrix gels. Biomaterials.

[196] Xu M., West-Farrell E.R., Stouffer R.L., Shea L.D., Woodruff T.K., Zelinski M.B. (2009). Encapsulated three-dimensional culture supports development of nonhuman primate secondary follicles. Biol. Reprod..

[197] King S.M., Quartuccio S., Hilliard T.S., Inoue K., Burdette J.E. (2011). Alginate hydrogels for three-dimensional organ culture of ovaries and oviducts. J. Vis. Exp..

[198] Jiang T., Munguia-Lopez J.G., Flores-Torres S., Kort-Mascort J., Kinsella J.M. (2019). Extrusion bioprinting of soft materials: An emerging technique for biological model fabrication. Appl. Phys. Rev..

[199] Wang J., Fu W., Zhang D., Yu X., Li J., Wan C. (2010). Evaluation of novel alginate dialdehyde cross-linked chitosan/calcium polyphosphate composite scaffolds for meniscus tissue engineering. Carbohyd. Polym..

[200] Xu Y., Huang C., Li L., Yu X., Wang X., Peng H., Gu Z., Wang Y. (2013). In vitro enzymatic degradation of a biological tissue fixed by alginate dialdehyde. Carbohyd. Polym..

[201] Kim W.S., Mooney D.J., Arany P.R., Lee K., Huebsch N., Kim J. (2012). Adipose tissue engineering using injectable, oxidized alginate hydrogels. Tissue Eng..

[202] Lin Y.H., Lee A.K.X., Ho C.C., Fang M.J., Kuo T.Y., Shie M.Y. (2022). The effects of a 3D-printed magnesium-/strontium-doped calcium silicate scaffold on regulation of bone regeneration via dual-stimulation of the AKT and WNT signaling pathways. Biomater. Adv..

[203] Augustine R. (2018). Skin bioprinting: A novel approach for creating artificial skin from synthetic and natural building blocks. Prog. Biomater..

[204] Liu P., Shen H., Zhi Y., Si J., Shi J., Guo L., Shen S.G. (2019). 3D bioprinting and in vitro study of bilayered membranous construct with human cells-laden alginate/gelatin composite hydrogels. Colloids Surf..

[205] Cheng L., Yao B., Hu T., Cui X., Shu X., Tang S., Wang R., Wang Y., Liu Y., Song W. (2019). Properties of an alginate-gelatin-based bioink and its potential impact on cell migration, proliferation, and differentiation. Int. J. Biol. Macromol..

[206] Wang S., Xiong Y., Chen J., Ghanem A., Wang Y., Yang J., Sun B. (2019). Three-dimensional printing bilayer membrane scaffold promotes wound healing. Front. Bioeng. Biotechnol..

[207] Somasekharan L.T., Raju R., Kumar S., Geevarghese R., Nair R.P., Kasoju N., Bhatt A. (2021). Biofabrication of skin tissue constructs using alginate, gelatin and diethylaminoethyl cellulose bioink. Int. J. Biol. Macromol..

[208] Zou Q., Tian X., Luo S., Yuan D., Xu S., Yang L., Ma M., Ye C. (2021). Agarose composite hydrogel and PVA sacrificial materials for bioprinting large-scale, personalized face-like with nutrient networks. Carbohyd. Polym..

[209] Masri S., Zawani M., Zulkiflee I., Salleh A., Fadilah N.I.M., Maarof M., Wen A.P.Y., Duman F., Tabata Y., Aziz I.A. (2022). Cellular interaction of human skin cells towards natural bioink via 3D-bioprinting technologies for chronic wound: A comprehensive review. Int. J. Mol. Sci..

[210] Liu J., Zhou Z., Zhang M., Song F., Feng C., Liu H. (2022). Simple and robust 3D bioprinting of full-thickness human skin tissue. Bioengineered.

[211] Silva L.P. (2019). Current trends and challenges in biofabrication using biomaterials and nanomaterials: Future perspectives for 3D/4D bioprinting. 3D and 4D Printing in Biomedical Applications.

[212] Liu Y., Hsu S.H. (2020). Biomaterials and neural regeneration. Neural Regen. Res..

[213] Guan S., Li J., Zhang K., Li J. (2021). Environmentally responsive hydrogels for repair of cardiovascular tissue. Heart Fail. Rev..

[214] Ostrovidov S., Salehi S., Costantini M., Suthiwanich K., Ebrahimi M., Sadeghian R.B., Fujie T., Shi X., Cannata S., Gargioli C. (2019). 3D bioprinting in skeletal muscle tissue engineering. Small.

[215] Lian Q., Zhou L., Li X., Mao W., Li D. (2020). Perfusive and osmotic capabilities of 3D printed hollow tube for fabricating large-scaled muscle scaffold. Rapid Prototyp. J..

[216] Sun Q., Silva E.A., Wang A., Fritton J.C., Mooney D.J., Schaffler M.B., Grossman P.M., Rajagopalan S. (2010). Sustained release of multiple growth factors from injectable polymeric system as a novel therapeutic approach towards angiogenesis. Pharm. Res..

[217] Borselli C., Storrie H., Benesch-Lee F., Shvartsman D., Cezar C., Lichtman J.W., Vandenburgh H.H., Mooney D.J. (2010). Functional muscle regeneration with combined delivery of angiogenesis and myogenesis factors. Proc. Natl. Acad. Sci. USA.

[218] Cattelan G., Guerrero Gerbolés A., Foresti R., Pramstaller P.P., Rossini A., Miragoli M., Caffarra Malvezzi C. (2020). Alginate formulations: Current developments in the race for hydrogel-based cardiac regeneration. Front. Bioeng. Biotechnol..

[219] Tous E., Purcell B., Ifkovits J.L., Burdick J.A. (2011). Injectable acellular hydrogels for cardiac repair. J. Cardiovasc. Transl. Res..

[220] Chiu L.L., Radisic M., Vunjak-Novakovic G. (2010). Bioactive scaffolds for engineering vascularized cardiac tissues. Macromol. Biosci..

[221] Ruvinov E., Leor J., Cohen S. (2011). The promotion of myocardial repair by the sequential delivery of IGF-1 and HGF from an injectable alginate biomaterial in a model of acute myocardial infarction. Biomaterials.

[222] Lv K., Li Q., Zhang L., Wang Y., Zhong Z., Zhao J., Lin X., Wang J., Zhu K., Xiao C. (2019). Incorporation of small extracellular vesicles in sodium alginate hydrogel as a novel therapeutic strategy for myocardial infarction. Theranostics.

[223] Levit R.D., Landázuri N., Phelps E.A., Brown M.E., García A.J., Davis M.E., Joseph G., Long Jr R., Safley S.A., Suever J.D. (2013). Cellular encapsulation enhances cardiac repair. J. Am. Heart Assoc..

[224] Feng J., Wu Y., Chen W., Li J., Wang X., Chen Y., Yu Y., Shen Z., Zhang Y. (2020). Sustained release of bioactive IGF-1 from a silk fibroin microsphere-based injectable alginate hydrogel for the treatment of myocardial infarction. J. Mater. Chem..

[225] Deng B., Shen L., Wu Y., Shen Y., Ding X., Lu S., Jia J., Qian J., Ge J. (2015). Delivery of alginate-chitosan hydrogel promotes endogenous repair and preserves cardiac function in rats with myocardial infarction. J. Biomed. Mater. Res. Part A.

[226] Rufaihah A.J., Seliktar D. (2016). Hydrogels for therapeutic cardiovascular angiogenesis. Adv. Drug Deliv. Rev..

[227] Ungerleider J.L., Christman K.L. (2014). Concise review: Injectable biomaterials for the treatment of myocardial infarction and peripheral artery disease: Translational challenges and progress. Stem Cells Transl. Med..

[228] Maiullari F., Costantini M., Milan M., Pace V., Chirivì M., Maiullari S., Rainer A., Baci D., Marei H.E.S., Seliktar D. (2018). A multi-cellular 3D bioprinting approach for vascularized heart tissue engineering based on HUVECs and iPSC-derived cardiomyocytes. Sci. Rep..

[229] Skjåk-Bræk G., Espevik T. (1996). Application of alginate gels in biotechnology and biomedicine. Carbohydr. Eur..

[230] Nayak A.K., Hasnain M.S., Aminabhavi T.M. (2021). Drug delivery using interpenetrating polymeric networks of natural polymers: A recent update. J. Drug Deliv. Sci. Technol..

[231] Singh S., Chunglok W., Nwabor O.F., Ushir Y.V., Singh S., Panpipat W. (2022). Hydrophilic biopolymer matrix antibacterial peel-off facial mask functionalized with biogenic nanostructured material for cosmeceutical applications. J. Polym. Environ..

[232] Mohapatra S., Mirza M., Hilles A.R., Zakir F., Gomes A.C., Ansari M.J., Iqbal Z., Mahmood S. (2021). Biomedical application, patent repository, clinical trial, and regulatory updates on hydrogel: An extensive review. Gels.

